# Spatial enhancer activation influences inhibitory neuron identity during mouse embryonic development

**DOI:** 10.1038/s41593-024-01611-9

**Published:** 2024-03-25

**Authors:** Elena Dvoretskova, May C. Ho, Volker Kittke, Florian Neuhaus, Ilaria Vitali, Daniel D. Lam, Irene Delgado, Chao Feng, Miguel Torres, Juliane Winkelmann, Christian Mayer

**Affiliations:** 1 Max Planck Institute for Biological Intelligence, Martinsried, Germany; 2 Max Planck Institute of Neurobiology, Martinsried, Germany; 3 Institute of Neurogenomics, Helmholtz Zentrum München GmbH, German Research Center for Environmental Health, Neuhererg, Germany; 4 TUM School of Medicine and Health, Institute of Human Genetics, Technical University of Munich, Munich, Germany; 5 DZPG (German Center for Mental Health), Munich, Germany; 6 Cardiovascular Development Program, Centro Nacional de Investigaciones Cardiovasculares (CNIC), Madrid, Spain; 7 Centro de Investigación Biomédica en Red de Enfermedades Cardiovasculares (CIBERCV), Madrid, Spain; 8 Departamento de Genética, Fisiología y Microbiología, Facultad de Biología, Universidad Complutense de Madrid, Madrid, Spain; 9 Munich Cluster for Systems Neurology (SyNergy), Munich, Germany

**Keywords:** Genetics of the nervous system, Cell fate and cell lineage

## Abstract

The mammalian telencephalon contains distinct GABAergic projection neuron and interneuron types, originating in the germinal zone of the embryonic basal ganglia. How genetic information in the germinal zone determines cell types is unclear. Here we use a combination of in vivo CRISPR perturbation, lineage tracing and ChIP–sequencing analyses and show that the transcription factor MEIS2 favors the development of projection neurons by binding enhancer regions in projection-neuron-specific genes during mouse embryonic development. MEIS2 requires the presence of the homeodomain transcription factor DLX5 to direct its functional activity toward the appropriate binding sites. In interneuron precursors, the transcription factor LHX6 represses the MEIS2–DLX5-dependent activation of projection-neuron-specific enhancers. Mutations of *Meis2* result in decreased activation of regulatory enhancers, affecting GABAergic differentiation. We propose a differential binding model where the binding of transcription factors at *cis*-regulatory elements determines differential gene expression programs regulating cell fate specification in the mouse ganglionic eminence.

## Abstract (web summary)

How genetic information in the germinal zone determines neuronal cell types is unclear. Here the authors show that MEIS2 plays an important role in determining GABAergic neuron diversity during development.

## Main

The ganglionic eminence (GE) is an embryonic subpallial structure which gives rise to various inhibitory GABAergic cell types in the forebrain. It is divided into the medial (MGE), caudal (CGE) and lateral (LGE) GEs. Each region creates non-overlapping types of GABAergic projections or interneurons (INs)^1^.

Several transcription factors (TFs) and their cofactors have been shown to be necessary for the specification of GABAergic subtypes^2^ and their dysregulation results in disease^2,3^. For example, members of the DLX family are present in the GE and are required for the development of GABAergic neurons^4^ and MEIS2, a member of the TALE family of homeodomain-containing TFs, has been implicated in the generation of LGE-derived GABAergic projection neurons (PNs)^5^. The mechanisms by which these TFs select and activate their lineage-specific target genes remain unclear.

Here we used sparse CRISPR–Cas-mediated perturbation of developmental TFs in GABAergic progenitors and tracked their developmental trajectories with lineage barcodes and single-cell RNA sequencing (scRNA-seq). We found that the sparse perturbation of *Meis2* increased the proportion of IN clones at the expense of PN clones and that MEIS2 requires the presence of the homeodomain TF DLX5 to direct its functional activity toward genomic binding sites of *cis*-regulatory elements (CREs) or enhancers associated with PN lineage-specific genes. A mutation of *Meis2* that causes intellectual disability in humans^6,7^ was much less able to potentiate the DLX5-induced activation of these CREs. Our results indicate that MEIS2 acts as a transcriptional activator to generate patterns of CRE activation which specify PN identities in GABAergic precursor cells. This mechanism may contribute to neurological dysfunction in diseases caused by *Meis2* mutations.

## Results

### Perturbation of *Meis2* alters the proportion of PNs and INs

**Fig. 1:**
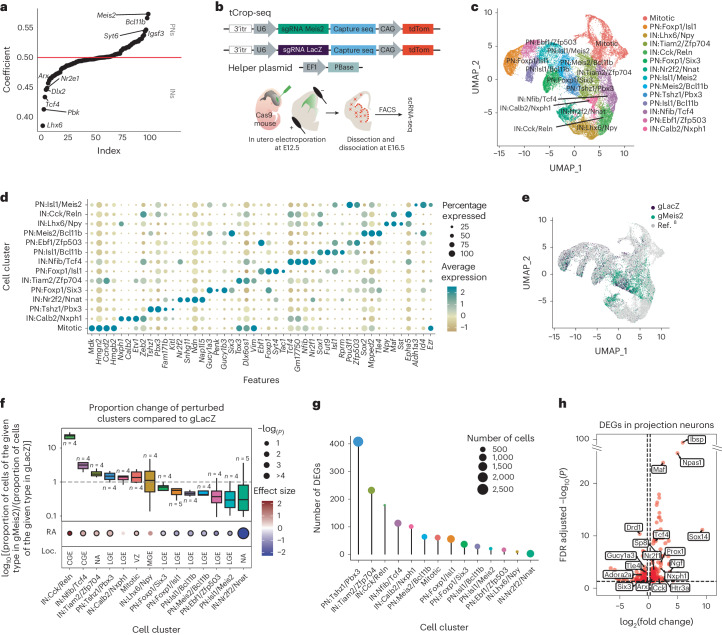
In vivo tCROP-seq of *Meis2* in the mouse forebrain. **a**, Logistic regression coefficients of genes being predictive of IN or PN fate. Genes with coefficients >0.5 are predictive of PN fate and genes with coefficients <0.5 are predictive of IN fate. Logistic regression model was trained on equal cell numbers for INs and PNs (*n* = 8,825). **b**, Vector maps and schematic of the in vivo tCROP-seq workflow in which mutations are introduced by in utero electroporation of sgRNAs and the effect is determined at a later time point by scRNA-seq. **c**, UMAP plot of inhibitory cells colored by clusters (*n* = 34,619 cells). **d**, Dotplot of the top four marker genes of inhibitory clusters. **e**, UMAP plot of the integrated dataset colored by sgRNAs. Gray dots represent cells from a published dataset (*n* = 21,454 cells from ref. ^8^; *n* = 13,165 cells from this study). **f**, Top, relative increase or decrease in the cell number in gMeis2 compared to gLacZ control in inhibitory neuron clusters. Plot shows log_10_((proportion of cells of the given type in gMeis2 perturbed animals)/(proportion of cells of the given type in gLacZ controls)). Bottom, perturbation effects in different clusters compared to gLacZ controls. Dot color corresponds to effect size, dot size corresponds to −log_10_(*P*). *P* values were derived from Poisson regression models. FDR correction was applied to the *P* values. The black outline indicates statistical significance (*P* ≤ 0.05). RE, regression analysis; Loc., location of the presumed origin of the cluster within the GE. Box plots show the median (center line), quartiles (box bounds), extend to 1.5 times the interquartile range (whiskers); *n* is the number of independent experiments (shown on the plot); color depicts clusters. NA, not assigned. **g**, Plot showing the distribution of DEGs between gMeis2 and gLacZ across various cell types. The height of each bar represents the number of DEGs in a specific cell type and the size of the dots is scaled on the basis of the number of cells in each type. Dot color depicts clusters. **h**, Volcano plot showing the results of the differential gene expression analysis in PNs at E16 (Methods). The *x* axis represents log_2_(fold change), the *y* axis represents the FDR adjusted −log_10_(*P*). The dotted lines show a cutoff (adjusted *P* ≤ 0.05, log_2_(fold change) <−0.3 and >0.3). Source data

**Extended Data Fig. 1:**
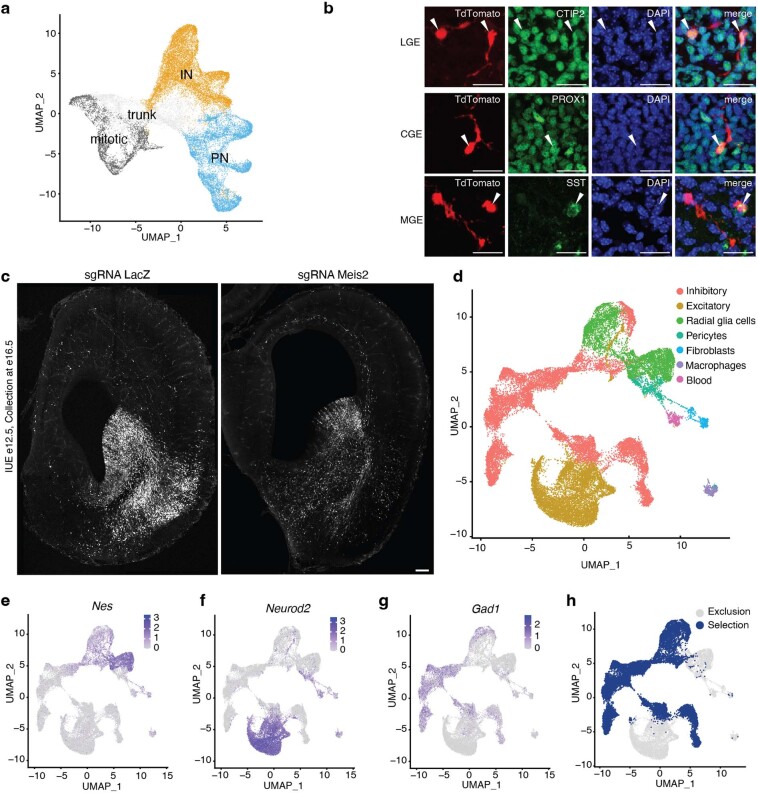
Targeting of sgRNAs to progenitors in the ganglionic eminence. **a**, UMAP depicting groups of cells used for a logistic regression analysis to predict PN and interneuron fate genes (n=29380 cells). Data from Bandler et al. (2022). **b**, Immunohistochemistry of E18.5 brains electroporated with tCROP-seq LacZ sgRNA vector at E12.5. Subsets of tdTomato expressing neurons show immunoreactivity against markers of different inhibitory neuron types: anti-CTIP2, LGE-derived striatal PNs; anti-SST, MGE-derived cortical INs; anti-PROX1, CGE-derived cortical INs. Coronal forebrain sections of 3 different mice were analysed. Scale bars = 20 *μ*m. **c**, Localization of tdTomato expression driven by gLacZ and gMeis2 plasmids in the cortex, striatum and GE at E16.5, following IUE at E12.5. Scale bar, 0.1 mm. 3 mice for each condition were analysed. **d**, UMAP plot displaying the E16 data colored by cell class (Inhibitory: 16098 neurons; Excitatory: 10010 neurons; Radial Glial: 5915 cells; Pericytes: 1008 cells; Fibroblasts: 537 cells; Macrophages: 523 cells; Blood: 390 cells) **e-g**, Feature plots depicting the expression of the canonical marker genes *Nes, Neurod2* and *Gad1* (n=34481 cells). **h**, UMAP plot illustrating the selection of cells for downstream analysis.

We conducted a logistic regression analysis on scRNA-seq data from the GE^8^ to identify regulatory TFs that play a role in determining the fate of GABAergic PNs or INs. Our findings revealed *Meis2* as the gene with the highest predictability for a PN fate, while *Lhx6* and *Tcf4* emerged as strong predictors of an IN fate (Fig. 1a and Extended Data Fig. 1a). To assess the effects of *Meis2* depletion on cell fate in a sparse population of GE precursors, we modified CROP-seq^9^, a method which integrates CRISPR–Cas perturbations with scRNA-seq readout. Specifically, we implemented a *piggyBac* transposon-based strategy (tCROP-seq) to increase the in vivo efficiency and to be able to deliver single-guide RNAs (sgRNAs) via in utero electroporation. tCROP-seq sgRNA vectors also encode tdTomato to enable the labeling and enrichment of perturbed neurons. Before conducting the tCROP-seq experiments, we validated the efficiency of the *Meis2* sgRNA in inducing frame-shift mutations both in vitro and in vivo (Supplementary Table 1).

The tCROP-seq vectors were targeted by in utero electroporation at E12.5 to progenitor cells of the GE in a mouse line ubiquitously expressing Cas9 (ref. ^10^) (Fig. 1b). At E16.5, most tdTomato+ cells had migrated away from the ventricular zone and colonized various structures, including the striatum, cerebral cortex and olfactory bulb (Extended Data Fig. 1b,c), consistent with the migration patterns of GE-derived inhibitory neurons at this stage^11^. Both immunohistochemical analysis of tdTomato+ cells at E18 and scRNA-seq analysis at E16 indicated that the tCROP-seq vectors were expressed across various MGE-, CGE- and LGE-derived inhibitory neuron types (see below).

For the tCROP-seq experiment, we collected a total of 14 embryos from 10 pregnant females (Supplementary Table 2). Of these, eight received sgRNAs for *Meis2* (gMeis2) and six received sgRNAs for *LacZ* (gLacZ), which served as a control. Cortices, striata and olfactory bulbs were dissected at E16 and tdTomato+ cells were enriched by FACS. To minimize batch effects, we pooled cells from embryos which received either gLacZ or gMeis2 and then performed multiplexed scRNA-seq (Fig. 1b; Methods). We sequenced six independent scRNA-seq experiments. Together, this resulted in a dataset containing 34,481 cells passing quality controls and filtering, which were linked with either gLacZ (11,009 cells) or gMeis2 (23,472 cells). We projected cells into a shared embedding using Harmony^12^ and applied a standard Seurat^13^ analysis pipeline (Extended Data Fig. 1d).

Louvain clustering grouped radial glia cells, excitatory neurons and inhibitory neurons into several clusters (Extended Data Fig. 1d). We subset cells from the inhibitory clusters where a gRNA could be recovered (13,165 inhibitory cells; Extended Data Fig. 1e–h) and integrated them with published scRNA-seq datasets from embryonic wild-type mice^8^, to get a higher resolution of inhibitory cell states (Fig. 1c–e). We annotated 14 clusters on the basis of shared marker gene expression and grouped them into three main classes: mitotic (mitotic), GABAergic PNs (PN:Foxp1/Six3, PN:Foxp1/Isl1, PN:Isl1/Bcl11b, PN:Ebf1/Zfp503, PN:Meis2/Bcl11b, PN:Isl1/Meis2 and PN:Tshz1/Pbx3) and GABAergic INs (IN:Calb2/Nxph1, IN:Tiam2/Zfp704, IN:Nfib/Tcf4, IN:Lhx6/Npy, IN:Cck/Reln and IN:Nr2f2/Nnat). Cells expressing gMeis2 contained a reduced proportion of PN cell types and an increased proportion of IN cell types, when compared to gLacZ controls (Fig. 1e,f). The proportion of CGE-derived IN populations was increased in the gMeis2 condition and the relative proportion of several PN types was decreased. This suggests that, under normal conditions, MEIS2 promotes the generation of LGE-derived PN types.

**Extended Data Fig. 2:**
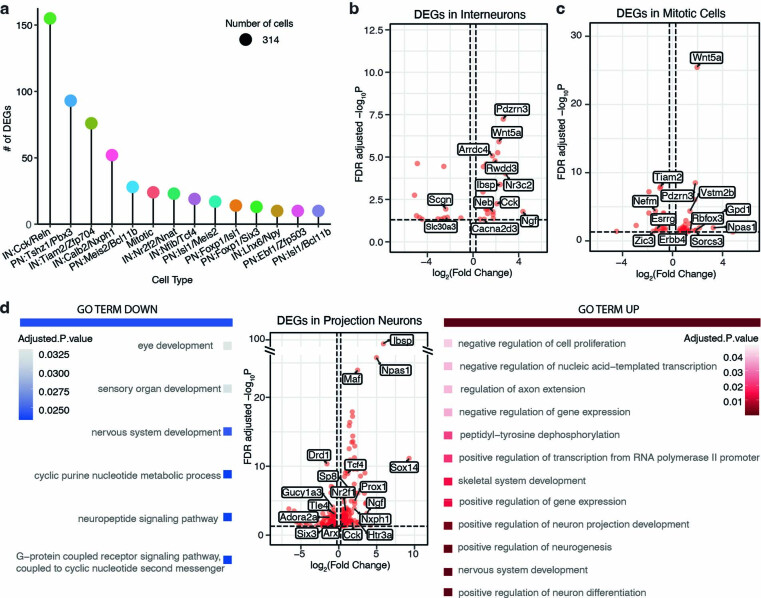
gMeis2 modulates gene expression in inhibitory neuron precursor cells. **a**, Lollipop plots illustrating the impact of gMeis2 on inhibitory clusters, with the number of DEGs shown after downsampling each group to 314 cells. **b**, Volcano plot depicting the DEGs in interneurons for gMeis2 compared to gLacZ. **c**, Volcano plot depicting the DEGs in mitotic cells for gMeis2 compared to gLacZ. **d**, Volcano plot depicting the DEGs in cells belonging to the projection neuron class for gMeis2 compared to gLacZ. Gene ontology analysis was performed separately on upregulated and downregulated DEGs. Significantly enriched GO terms are shown (adjusted *P* value ≤0.05). In **b**,**c**,**d**, the dotted lines depict a cutoff (FDR *P* value ≤0.05, log_2_(Fold Change) <-0.3 and >0.3). Source data

The impact of gMeis2 on differential gene expression was strongest on the clusters PN:Tshz1/Pbx3, IN:Tiam2/Zfp704 and IN:Cck/Reln (Fig. 1g and Extended Data Fig. 2a). In PN clusters, gMeis2+ cells showed decreased expression levels of genes known to be associated with PN identity, such as *Adora2a*, *Drd1* and *Six3* (refs. ^14,15^), compared to gLacZ. Many genes related to IN development and specification, such as *Maf*, *Tcf4*, *Prox1* and *Arx*^16–18^, were upregulated in PN clusters (Fig. 1h). Furthermore, the proportion of mitotic progenitors was increased in gMeis2 compared to gLacZ. Genes involved in cell proliferation and differentiation were upregulated in the mitotic cluster in gMeis2, in particular the gene *Wnt5a*, which is part of the non-canonical WNT signaling pathway^19^ (Fig. 1f and Extended Data Fig. 2b,c). Gene ontology enrichment analysis of differentially expressed genes (DEGs) in PNs showed that processes such as neuron development, axon extension and neuron differentiation were deregulated in the gMeis2 condition (Extended Data Fig. 2d).

### MEIS2 depletion shifts the clonal composition of precursors

**Fig. 2:**
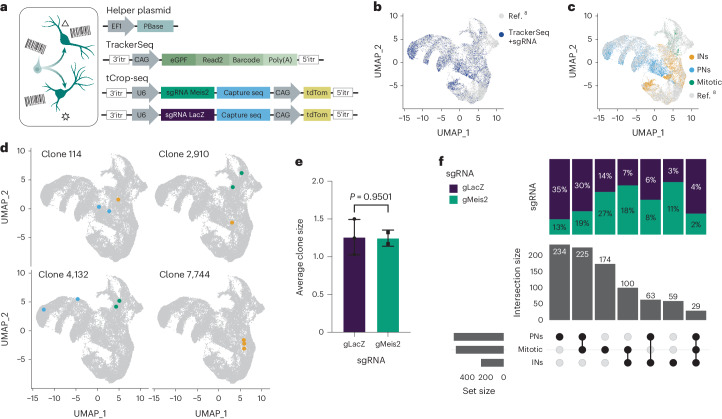
In vivo TrackerSeq lineage tracing and tCROP-seq perturbation of *Meis2*. **a**, Schematic of TrackerSeq lineage tracing, in which clonal boundaries are determined using heritable RNA tags. **b**, UMAP of the integrated dataset with labeling of cells containing TrackerSeq lineage barcodes (*n* = 13,165 cells where a gRNA and TrackerSeq lineage barcode could be recovered; *n* = 21,454 cells from ref. ^8^). **c**, UMAP of the integrated dataset colored by cell class (mitotic, INs and PNs; *n* = 34,619 cells). **d**, Examples of clones which are shared between classes and an example of a clone restricted to one class (*n* = 34,619 cells per plot). **e**, Clone sizes for gLacZ and gMeis2. Bars, mean ± s.d. The dots represent the mean number of clones for each gRNA in each independent experiment (*n* = 3 experiments for gLacZ and *n* = 2 experiments for gMeis2). Statistical significance was assessed by two-way ANOVA, *P* = 0.9825. **f**, UpSet plot showing clonal intersections between groups of clusters. The bar graph on top shows the proportion of clones belonging to gLacZ or gMeis2. The bar graph in the middle shows the number of observed intersections. The bar graph on the left indicates the number of cells per group. Source data

**Extended Data Fig. 3:**
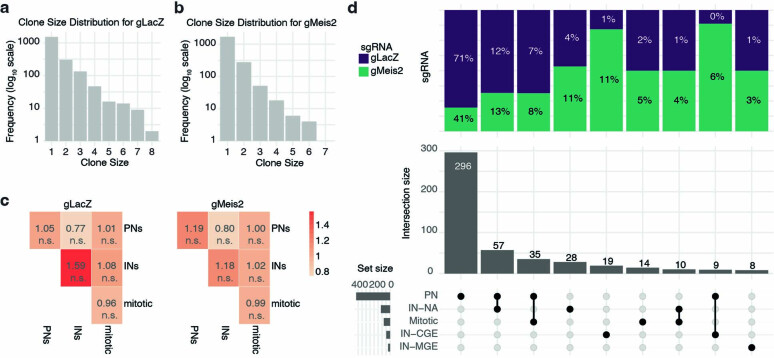
Combined lineage tracing and CRISPR-Cas9 induced perturbation. **a**, Histogram depicting the clone size distribution for gLacZ. **b**, Histogram depicting the clone size distribution for gMeis2. **c**, Clonal coupling between cell states. The number of shared barcodes between pairs of cell types was normalized by the expectation when clonal membership is shuffled. Normalized metrics close to 1 indicate that clonal coupling is consistent with random expectation. The observed trend did not pass the statistical threshold of empirical FDR-corrected *P* values of ≤0.05; see Methods; ns, not significant. **d**, UpSet plot showing clonal intersections between cell classes. The bar graph on top displays the proportion of clones belonging to gLacZ or gMeis2. The bar graph in the middle shows the number of observed intersections. The bar graph on the left indicates the number of cells per group. Mitotic (mitotic progenitors); PN (projection neuron precursors); IN-CGE (CGE interneuron precursors); IN-MGE (MGE-interneuron precursors); and IN-NA (interneurons from intermediate clusters that we couldn’t assign to either MGE or CGE; Classification into categories was made based on the cluster annotations, see Fig. 1f, Supplementary Table 3). Source data

To investigate whether MEIS2 depletion in LGE-PN precursors may switch the fate of their progeny to a CGE/MGE-INs identity, we combined tCROP-seq with TrackerSeq^8^. TrackerSeq integrates heritable DNA barcodes into electroporated progenitors, allowing tracking clonal relationships between their daughter neurons (Fig. 2a). The tCROP-seq and TrackerSeq can be used simultaneously because we have implemented a similar transposase strategy for both methods. We used in utero electroporation at E12.5 to introduce the TrackerSeq barcode library and tCROP-seq sgRNAs to cycling progenitors in the GE. At E16.5, we collected tdTomato–EGFP+ cells from four independent batches and prepared sequencing libraries for transcriptomes, sgRNAs and lineage barcodes. The cells with TrackerSeq barcodes were part of the preceding tCROP-seq analysis and were thus integrated in the same embedding (Fig. 2b). Consistent with ref. ^8^, we found clones composed of mitotic cells, PNs, INs and combinations thereof (Fig. 2c,d). The average clonal size of multicell in gMeis2 was similar compared to gLacZ (Fig. 2e and Extended Data Fig. 3a,b), suggesting that cell cycle dynamics or cell death are unlikely to account for the proportional shift in cell fate. The proportion of clones consisting of only mitotic cells was increased in gMeis2 compared to gLacZ, which agrees with a report showing that MEIS2 is required for LGE progenitors to leave the cell cycle^5^. When we compared clonal patterns of gMeis2 and gLacZ cells, we observed a shift toward IN-only and mitotic-IN clones. Conversely, the number of PN-only and mitotic-PN clones was decreased (Fig. 2f). Furthermore, the coupling of multicell clones within IN clusters (Methods) showed a tendency to decrease in gMeis2, although this did not pass statistical thresholds. This may suggest a broader range of lineages developing into IN precursors as a result of the fate switch from PNs to INs. (Extended Data Fig. 3c). To test whether there are differential effects in how gMeis2 affects CGE-like and MGE-like populations of IN precursors, we divided them on the basis of the cluster annotation (Fig. 1f) into CGE (IN-CGE) or MGE (IN-MGE) IN precursors (Extended Data Fig. 3d). In the gMeis2 condition, the number of PN clones was reduced and the number of both IN-CGE and IN-MGE clones was increased. Notably, in the gMeis2 condition, we observed several clones spreading across PN and IN-CGE, suggesting that progenitors that originally produced PNs switched to producing IN-CGE in the presence of gMeis2. Our results show that gMeis2 perturbation in progenitors leads to a partial shift in newly formed neurons from PN precursors to IN precursors.

### Genomic binding of DLX5 and MEIS2 in the embryonic GE

**Fig. 3:**
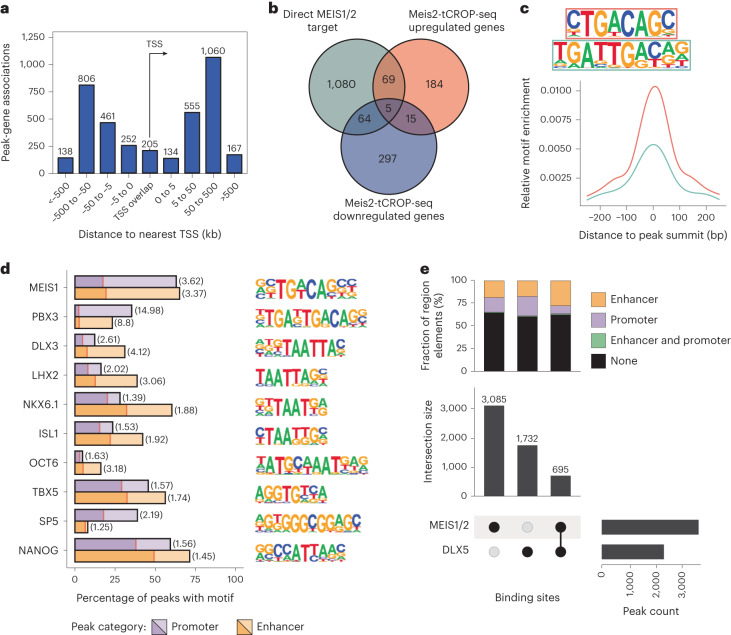
DNA-binding sites of MEIS1/2 in the GE at E14.5. **a**, Distribution of MEIS1/2 ChIP–seq peaks relative to the nearest TSS. **b**, Venn diagram showing overlap between MEIS1/2 target genes and genes upregulated or downregulated in inhibitory neurons of gMeis2 tCROP-seq. A cutoff was applied to select DEGs (adjusted *P* ≤0.05, log_2_(fold change) <−1.0 and >1.0). Overlap of upregulated and downregulated genes is due to opposite regulation in different subtypes of inhibitory neurons. **c**, De novo identified MEIS1/2-binding motifs and their position relative to peak summits. **d**, Motif occurrence of selected known motifs enriched within enhancer- or promoter-overlapping MEIS1/2-binding sites (light bars) compared to G/C-matched reference sequences (dark bars), with fold-enrichment in parentheses. **e**, Overlap between binding sites of MEIS1/2 and DLX5 (bottom), with respective distribution of binding sites overlapping promoter and/or enhancer regions. Source data

**Extended Data Fig. 4:**
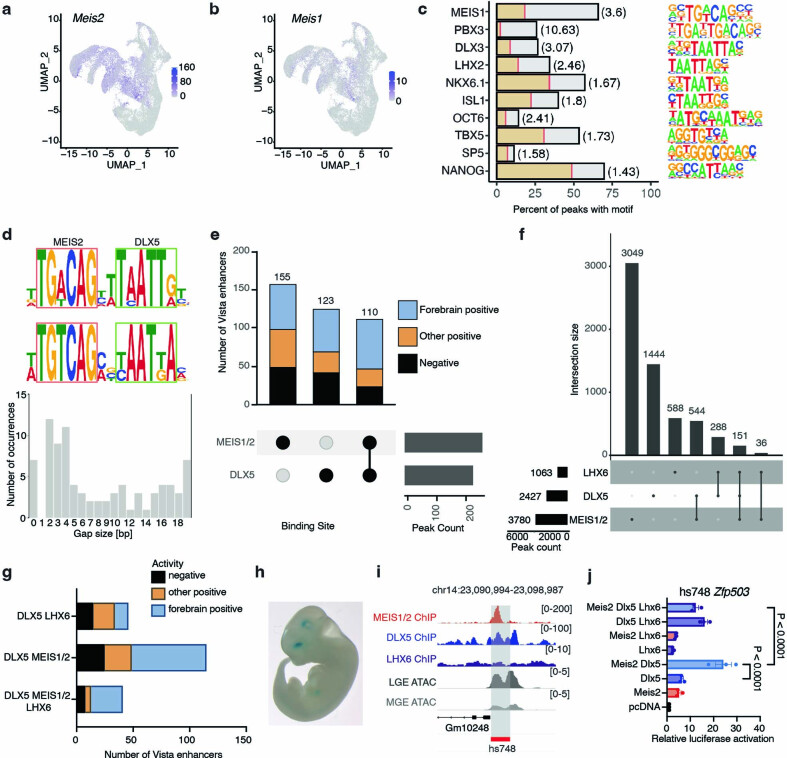
Overlap of MEIS1/2 and DLX5 ChIP–seq binding sites in the ganglionic eminence. **a**, Feature plot depicting the expression level of *Meis2* at E16 (n = 34619 cells). **b**, Feature plot depicting the expression level of *Meis1* at E16 (n = 34619 cells). **c**, Motif occurrence analysis of selected known motifs enriched within all MEIS1/2-binding sites (gray bars) compared to G/C-matched reference sequences (yellow). **d**, Motif spacing analysis of MEIS2 and DLX5 motifs within shared binding sites. The position weight matrix (PWM) of the most frequent motif configuration is shown on the left, while the right panel illustrates the overall distribution of the DLX5 motif in relation to the MEIS2 motif. **e**, Overlap analysis of binding sites between MEIS1/2 and DLX5 (bottom) and their distribution within different classes of Vista enhancers (top). **f**, Overlap analysis of binding sites between MEIS1/2, DLX5 and LHX6. **g**, Quantification of enhancers with MEIS1/2–DLX5-LHX6 overlapping peaks in respect to VISTA enhancers. 41 were within VISTA enhancers and 28 of these enhancers showed activity in the developing forebrain. **h**, Visualization of LacZ expression driven by the *hs748* enhancer in the E12.5 mouse forebrain Visel et al. (2007). **i**, Representative tracks of GE ChIP–seq of MEIS1/2 at E14.5 (red), DLX5 at E13.5 (blue) Lindtner et al. (2019), LHX6 at E13.5 (purple) Sandberg et al. (2016) and scATAC-seq Rhodes et al. (2022) from the LGE (dark gray) and MGE (gray) at E12.5. **j**, Overlap between binding sites of MEIS1/2, DLX5 and LHX6 in enhancer *hs748*, which associated with the gene *Zfp503*. **j**, Luciferase activity driven by *hs748*, cotransfected with Meis2, Dlx5 and Lhx6 expression vectors in Neuro2a cells. Bars represent mean ± s.e.m from a total of 9 replicates, split into three independent batches, each performed in triplicate. Points represent the mean of each batch for each condition. Statistical significance was assessed by two-way ANOVA. *P* values of pairwise comparisons from post hoc Tukey’s HSD are presented for selected conditions. For *P* values between specific conditions, see Source Extended Data for Fig. 4. Source data

To identify target genes of MEIS2, we performed chromatin immunoprecipitation followed by sequencing (ChIP–seq) on GE tissue dissected from E14.5 mouse embryos, using a combination of anti-MEIS1/2 and anti-MEIS2 antibodies. In the GE, the expression of *Meis2* is higher and more widespread than that of *Meis1*, therefore the antibodies are likely to bind primarily to MEIS2 epitopes (Extended Data Fig. 4a,b). We identified 3,780 MEIS1/2-binding sites, of which 16% were located within 5 kilobase (kb) of a transcription start site (TSS; Fig. 3a). Of the binding sites, 20% overlapped with developmental enhancers linked to putative target genes^20^ (Source Data for Fig. 3). Our data predict that MEIS1/2 directly regulates 1,218 target genes, either by binding to their TSS or to distal enhancers. Many of the target genes (11%) were up- or down-regulated in gMeis2 tCROP-seq positive PN clusters (Fig. 3b). De novo motif analysis showed the previously described MEIS1/2 core hexameric and decameric binding motifs TGACAG and TGATTGACAG, which were highly enriched at the centers of the peaks. These motifs correspond to either the binding of the MEIS homodimer or the MEIS–PBX heterodimer, respectively^21,22^ (Fig. 3c). Binding motifs containing the core sequence TAATT were strongly enriched in MEIS1/2 ChIP–seq peaks and enriched at enhancers compared to TSS-associated regions. This motif is shared by several homeodomain TF families including those of DLX, LHX and ISL (Fig. 3d and Extended Data Fig. 4c)^2^, of which several members are expressed in the GE^23,24^. Among them, we found the strongest enrichment for the binding motif of DLX3.

**Extended Data Fig. 5:**
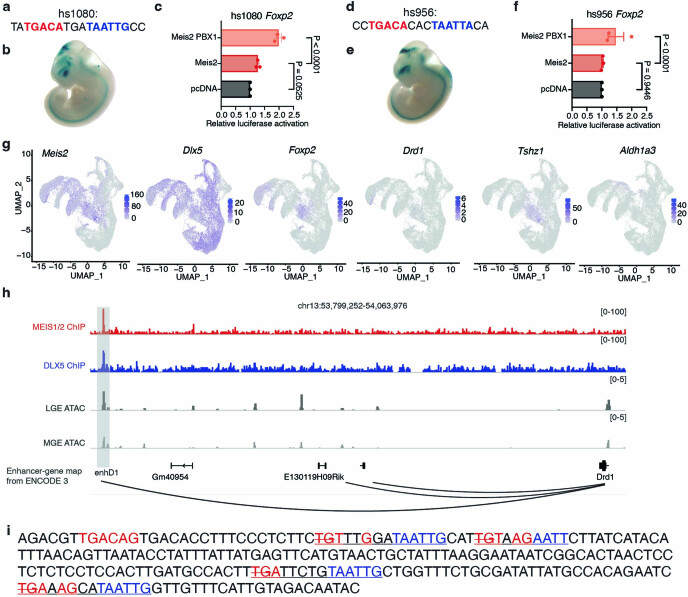
Regulation and functional analysis of PN gene enhancers. **a, d**, Combined MEIS (red) and DLX (blue) binding motifs found within *hs1080* (**a**) and *hs956* (**d**) enhancers. **b**, *hs1080* and **e**, *hs956* enhancers drive LacZ expression in the E12.5 mouse forebrain Visel et al. (2007). **c, f**, Luciferase assay measuring the activation effect of MEIS2 and PBX1 on *hs1080* (**c**) or *hs956* (**f**) driven luciferase reporter in Neuro2a cells. **g**, Feature plot depicting the expression level of *Meis2*, *Dlx5*, *Foxp2*, *Drd1*, *Tshz1* and *Aldh1a3* at E16 (n=34619 cells per plot). **h**, Visualization of the Drd1 locus with aligned tracks of MEIS1/2 ChIP–seq at E14.5 (red), DLX5 ChIP–seq at E13.5 (blue) and LGE (dark gray) Rhodes et al. (2022). The predicted enhancer-gene interactions are also depicted Gorkin et al. (2020). **i**, Depiction of the DNA sequence of the shortened version of the enhancer *enhD1*, highlighting the combined MEIS (red) and DLX (blue) binding motifs. The TG bases removed in the mutated version of *enhD1* are indicated with a strikeout line. In panels **c** and **f**, bars represent mean ± s.e.m from a total of 9 replicates, split into three independent batches, each performed in triplicate. Points represent the mean of each batch for each condition. Statistical significance was assessed by two-way ANOVA. *P* values of pairwise comparisons from post hoc Tukey’s HSD are presented for selected conditions. For *P* values between specific conditions, see Source Extended Data for Fig. 5. Source data

All DLX TFs share a common conserved motif, of which DLX1, DLX2, DLX5 and DLX6 are known to be master regulators of inhibitory neuron development in the forebrain^4^. Because *Meis2* and *Dlx5* are co-expressed in PN precursor cells of the LGE (Extended Data Fig. 5g), we compared the binding sites of MEIS1/2 with those of a published DLX5 ChIP–seq dataset in mouse GE^4^. Numerous MEIS1/2-binding sites (695; 18%) overlapped with DLX5-binding sites. The proportion of enhancers at shared (MEIS1/2–DLX5) binding sites was significantly increased compared to MEIS1/2- and DLX5-exclusive binding sites (Fig. 3e; *P* = 8.856 × 10^−9^, Chi^2^-test). The most common motif spacing was 2–4 base pairs (bp). In contrast to published in vitro experiments which observed a fixed spacing of 2 bp between MEIS1 and DLX3 (ref. ^25^), we observed a wider range of spacing (Extended Data Fig. 4d). Together, our findings suggest a potential cooperative role of MEIS1/2 and DLX5 in the fate determination of GE-derived neurons.

### Functional link between MEIS2–DLX5 and PN fate

**Fig. 4:**
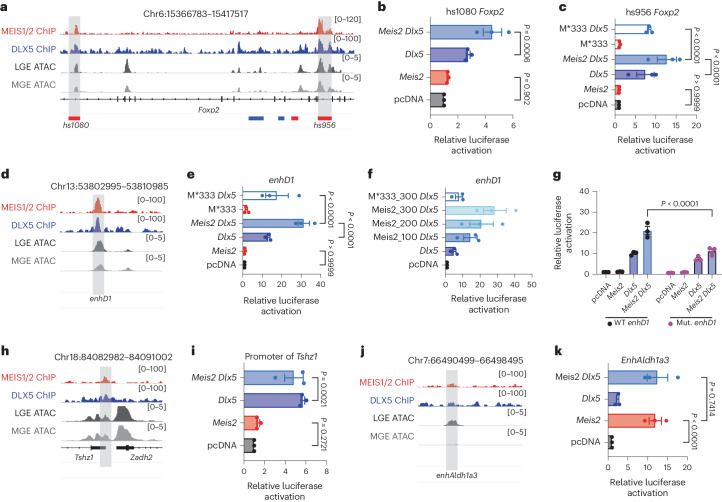
Cooperation between MEIS2 and DLX5 activates enhancers of projection-neuron-specific genes. **a**, Representative profiles of MEIS1/2 (red) and DLX5 (blue) ChIP–seq at E14.5 and E13.5, respectively, as well as scATAC-seq from LGE (dark gray) and MGE (gray) at E12.5 are shown at the *Foxp2* gene locus. DLX5 ChIP–seq data from ref. ^4^; scATAC-seq data from ref. ^27^. **b**, Luciferase activity driven by the enhancer *hs1080*, cotransfected with *Meis2* and *Dlx5* expression vectors in Neuro2a cells. **c**, Luciferase reporter assays of the enhancer *hs956*. **d**, Representative profiles of the *Drd1* gene enhancer *enhD1*. **e**, Luciferase reporter assays of *enhD1*. **f**, Luciferase reporter assays of *enhD1*, cotransfected with *Dlx5* and increasing concentration of *Meis2* or with Meis2*333. **g**, Luciferase reporter assays of the wild-type (WT) or mutated (mut.), shorter version of *enhD1*. **h**, Representative profiles of the *Tshz1* promoter. **i**, Luciferase reporter assays of the *Tshz1* promoter. **j**, Representative profiles of *Aldh1a3* enhancer *enhAldh1a3*. **k**, Luciferase reporter assays of *enhAldh1a3*. In **b**,**c**,**e**,**f**,**g**,**i** and **k**, bars represent mean ± s.e.m from a total of nine replicates, split into three independent batches, each performed in triplicate. Points represent the mean of each batch for each condition. Statistical significance was assessed by two-way ANOVA. *P* values of pairwise comparisons from post hoc Tukey’s HSD are presented for selected conditions. Exact *P* values between specific conditions are shown in Source Data for Fig. 4. Source data

To investigate the possibility of a functional link between MEIS2 and DLX5 in PN development, we performed a series of dual luciferase reporter assays to measure the activity of select enhancers in the presence of MEIS2, DLX5 or both. To select enhancers, we intersected MEIS1/2–DLX5 cobinding sites from ChIP–seq data with the VISTA in vivo enhancer database^26^ (Extended Data Fig. 4e). Additionally, we confirmed the accessibility of the respective genomic regions, using published scATAC-seq data of the LGE and MGE^27^. First, we chose two enhancers (*hs1080* and *hs956*) of the TF *Foxp2*, which both contained MEIS–DLX motifs with a spacing of 3 bp (ref. ^26^) (Fig. 4a and Extended Data Fig. 5a,b,d,e). *Foxp2* is expressed in precursors of GABAergic PNs (Extended Data Fig. 5g), has previously been implicated in PN development^28^ and is one of the genes that we found to be downregulated in gMeis2 tCROP-seq experiments (Source Data for Fig. 1). We transfected Neuro2a cells with a plasmid containing a selected enhancer upstream of a minimal promoter and the firefly luciferase gene, as well as a control plasmid encoding the NanoLuc luciferase gene under the PGK promoter. Additionally, we transfected the cells with plasmids encoding *Dlx5*, *Meis2* or both. MEIS2 alone did not significantly activate either enhancer and both *Foxp2* enhancers were only modestly activated in the presence of DLX5 alone (Fig. 4b,c). Together, MEIS2 and DLX5 potentiated the DLX5-induced activation of the *Foxp2* enhancers. As expected, PBX1, a known interaction partner of MEIS2 (ref. ^21^), increased the effect of MEIS2 (Extended Data Fig. 5c,f). These results suggest that MEIS2 and DLX5 bind cooperatively at specific binding sites of enhancers to regulate *Foxp2* expression. Mutations affecting a conserved amino acid (Arg333) of MEIS2 have been associated with severe intellectual disability^6,7^. We found that a missense variant (MEIS2*333, p.Arg333Lys) significantly reduced MEIS2–DLX5-dependent activation of the *Foxp2* enhancer *hs956* (Fig. 4c).

Next, we investigated whether the cooperation of MEIS2 and DLX5 at cobinding sites activates a putative regulatory enhancer (*enhD1*) of *Drd1*. *Drd1* encodes for the dopamine receptor D1, which is a marker of D1-type medium spiny PNs (D1-MSN; PN:Foxp1/Isl1, PN:Isl1/Bcl11b, PN:Ebf1/Zfp503) in the striatum^15^ (Supplementary Table 3). *Drd1* gene expression was strongly reduced in PN clusters in gMeis2 tCROP-seq experiments (Fig. 1h). The *enhD1* is predicted to be associated with *Drd1* (Extended Data Fig. 5h)^20^. Furthermore, *enhD1* contained pronounced ChIP–seq peaks for DLX5 and MEIS1/2 (Fig. 4d) and several MEIS–DLX cobinding motifs (Extended Data Fig. 5i). Similar to the *Foxp2* enhancers, MEIS2 did not activate *enhD1* but it potentiated the effect of DLX5, in a concentration-dependent manner (Fig. 4e,f). The cooperative activation of *enhD1* by MEIS2 and DLX5 was greatly reduced with the mutated version of MEIS2 (MEIS2*333). A truncated version of *enhD1* in which a portion (TG) of the MEIS-binding motif was removed at several sites of the enhancer (Extended Data Fig. 5i), showed reduced activation by MEIS2–DLX5 compared with the unmodified truncated *enhD1* (Fig. 4g). Taken together, our findings suggest that the cooperation of MEIS2 and DLX5 at specific cobinding sites within CREs activates projection-neuron-specific gene expression to promote PN fate.

**Extended Data Fig. 6:**
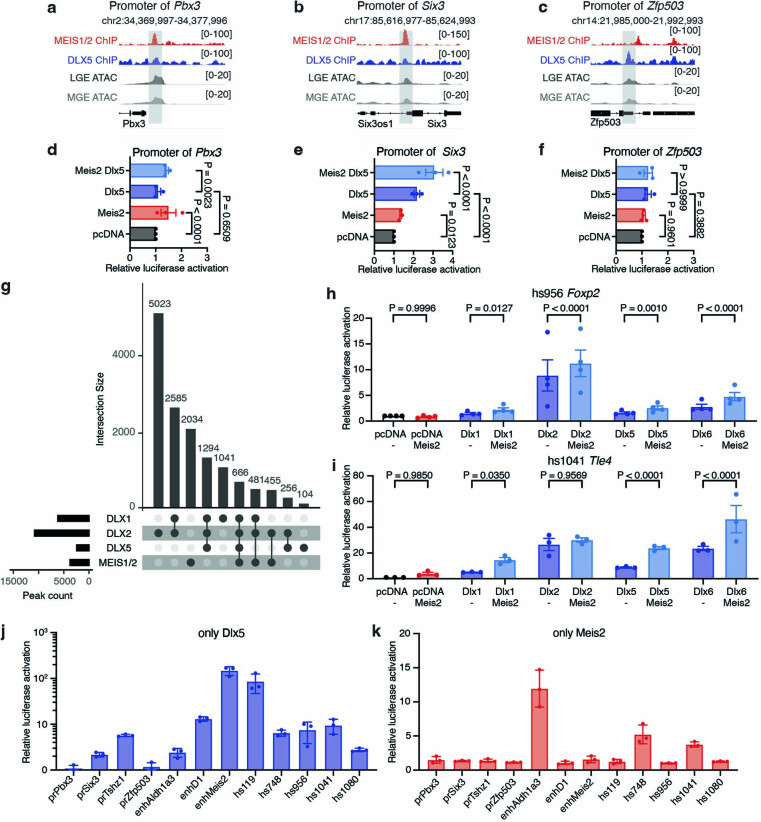
MEIS2 acts primarily via distal enhancers in the ganglionic eminence. **a-c**, Representative tracks of MEIS1/2 ChIP–seq in the GE at E14.5 (red), DLX5 ChIP–seq in the GE at E13.5 (blue) Lindtner et al. (2019) and scATAC-seq in the LGE (dark gray) and MGE (gray) at E12.5 Rhodes et al. (2022) are shown at the gene promotors of *Pbx3*, *Six3* and *Zfp503*. **d-f**, Luciferase activity driven by promoters of *Pbx3*, *Six3* and *Zfp503* genes, transfected with MEIS2 and DLX5 expression vectors in Neuro2a cells. **g**, Overlap between binding sites of MEIS1/2, DLX1, DLX2 and DLX5. **h-i**, Luciferase activity driven by the enhancer *hs956* (**j**) and *hs1080* (**k**), transfected with MEIS2 (g) and DLX1, DLX2, DLX5 or DLX6 expression vectors in Neuro2a cells. **j-k**, Luciferase activity driven by enhancers, transfected with MEIS2 (**j**) or DLX5 (**k**) expression vectors in Neuro2a cells. The data represents the combined results from multiple experiments. In panels **d, e, f, g, h, j** and **k**, bars represent mean ± s.e.m from a total of 9 or 12 replicates, split into 3–4 independent batches, each performed in triplicate. Points represent the mean of each batch for each condition. Statistical significance was assessed by two-way ANOVA. *P* values of pairwise comparisons from post hoc Tukey’s HSD are presented for selected conditions. For *P* values between specific conditions, see Source Extended Data for Fig. 6. Source data

Next, we tested whether MEIS2 can also activate the promoters of its target genes *Pbx3*, *Tshz1*, *Zfp503* and *Six3*. All three genes are marker genes for different PN clusters (Fig. 1d) and they all contain binding sites for MEIS in their promoters (Fig. 4h,i and Extended Data Fig. 6a–f). We found that the activation of these promoters by MEIS2 is small. Even the *Tshz1* promoter, which contains both DLX5 and MEIS1/2 motifs, was not activated by MEIS2, nor was MEIS2 able to enhance the DLX5-induced activation of this promoter. This may be because the motifs for MEIS1/2 are far away from DLXs motifs.

Our data suggest that in the GE, MEIS2 requires the presence of DLX5 to bind and co-activate enhancers with specific cobinding sites and this process induces gene expression related to PN development. We performed additional reporter assays, where we included additional members of the DLX family (DLX1, DLX2 and DLX6) and expanded the analysis of the ChIP–seq datasets to include DLX1 and DLX2. We found that MEIS2 can potentiate the activity of the tested enhancers in cooperation with DLX1/2/6 (Extended Data Fig. 6g–i).

We tested a total of eight enhancers of genes which are known to be important for inhibitory neuron development using the reporter assay (Extended Data Fig. 6j,k) and the results support this model. Of the enhancers tested, only the LGE-specific enhancer of *Aldh1a3*, *enhAldh1a3*, which lacks a MEIS1/2–DLX5 cobinding site, was strongly activated by MEIS2 alone (Fig. 4j,k). *Aldh1a3* encodes an enzyme that synthesizes retinoic acid in LGE precursors at E12.5 (refs. ^29,30^) and is essential for the differentiation of striatal PNs^31^. *Aldh1a3* was greatly downregulated in several clusters in the gMeis2 tCROP-seq experiments (Source Data for Fig. 1). It remains unclear whether MEIS2 is able to activate *enhAldh1a3* on its own or whether another cofactor, present in Neuro2a cells, is required.

### Spatial enhancer activation by MEIS2 and DLX5 in the LGE

**Extended Data Fig. 7:**
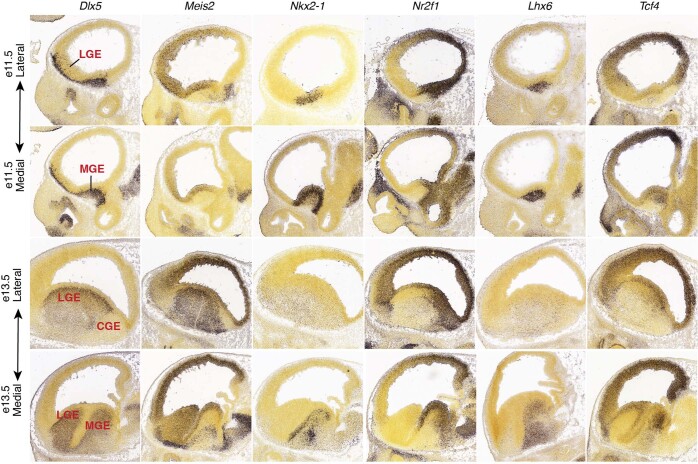
Transcription factor expression patterns in the ganglionic eminence. In situ Hybridization (ISH) images of *Dlx5*, *Meis2*, *Nkx2-1*, *Nr2f1*, *Lhx6* and *Tcf4* from the Allen Brain Institute’s Developing Mouse Brain Atlas at E11.5 and E13.5. MGE, medial ganglionic eminence; LGE, lateral ganglionic eminence; CGE, caudal ganglionic eminence.

PNs of the striatum originate largely in the LGE and many IN types, for example, those of the cortex, originate in the MGE and CGE^15,16,32^. *Meis2* messenger RNA is initially expressed broadly in the ventricular zone of the LGE, CGE and MGE. In neuronal precursors of the subventricular zone (SVZ) and mantle zone, a spatial pattern of *Meis2* expression emerges, where *Meis2* continues to be highly expressed in the LGE but is absent in the MGE (Extended Data Fig. 7)^5,30^.

**Fig. 5:**
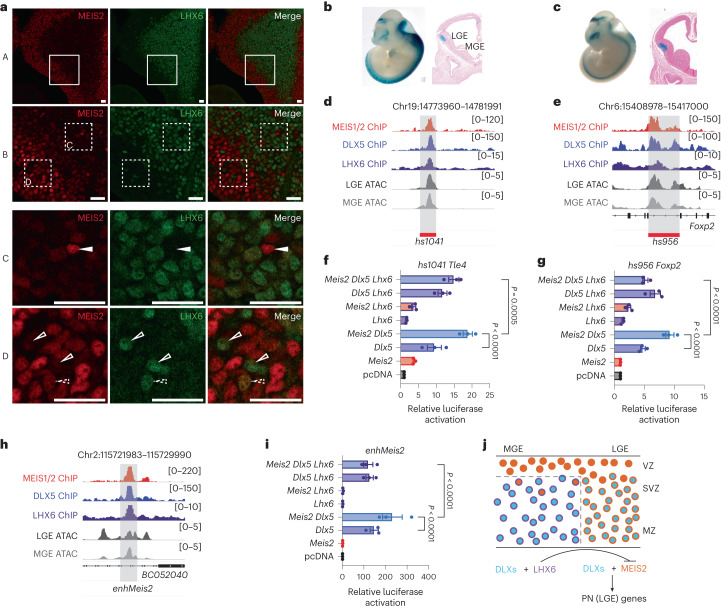
Regulation of LGE enhancers by MEIS2, DLX5 and LHX6. **a**, Immunohistochemistry of MEIS2 and LHX6 in the MGE of E13.5 embryos. MEIS2 immunoreactivity is high in cells of the ventricular zone (VZ) and low as cells transition to the mantle zone (MZ). Few cells in the SVZ retain MEIS2 expression (white triangle). Conversely, few cells in the VZ are immunoreactive for LHX6 (empty triangles). Some cells at the VZ to SVZ interface are co-immunoreactive against MEIS2 and LHX6 (dotted triangles). Coronal forebrain sections of three wild-type mice were analysed. Scale bars, 20 μm. **b**, LacZ expression in the LGE of E12.5 embryos, driven by the enhancer *hs1041* (ref. ^26^). **c**, LacZ expression in the LGE of E12.5 embryos, driven by the enhancer *hs9566*. **d**,**e**, Representative tracks of MEIS1/2 ChIP–seq in the GE at E14.5 (red), DLX5 ChIP–seq in the GE at E13.5 (blue)^4^, LHX6 ChIP–seq in the GE at E13.5 (purple)^35^ and scATAC-seq in LGE (dark gray) and MGE (gray) at E12.5 (ref. ^27^) are shown for the enhancers *hs1041* (**d**) and *hs956* (**e**). **f**,**g**, Luciferase activity driven by the enhancers *hs1041* (**f**) and *hs956* (**g**), cotransfected with *Meis2*, *Dlx5* and *Lhx6* expression vectors in Neuro2a cells. **h**, Representative tracks of enhancer *enhMeis2*. **i**, Luciferase reporter assays of *enhMeis2*. **j**, Model of the proposed actions of MEIS2, DLX5 and LHX6. MEIS2 promotes PN fate in the presence of DLX. LHX6 represses *Meis2* expression and function. SVZ, subventricular zone. In **f**–**i**, bars represent mean ± s.e.m from a total of nine replicates, split into three independent batches, each performed in triplicate. Points represent the mean of each batch for each condition. Statistical significance was assessed by two-way ANOVA. *P* values of pairwise comparisons from post hoc Tukey’s HSD are presented for selected conditions. For *P* values between specific conditions, see Source Data for Fig. 5. Source data

We next asked how the functional activity and expression of MEIS2 become LGE-specific, suspecting involvement of LHX6. This is supported by exclusive expression of *Lhx6* in the SVZ and mantle zone of MGE (contrasting with *Meis2*; Extended Data Fig. 7)^24^, with a small cell population at the ventricular zone and SVZ interface which showed MEIS2 and LHX6 co-immunoreactivity (Fig. 5a). Moreover, LHX6 is known to be vital for defining cortical IN subtypes^33–35^.

**Extended Data Fig. 8:**
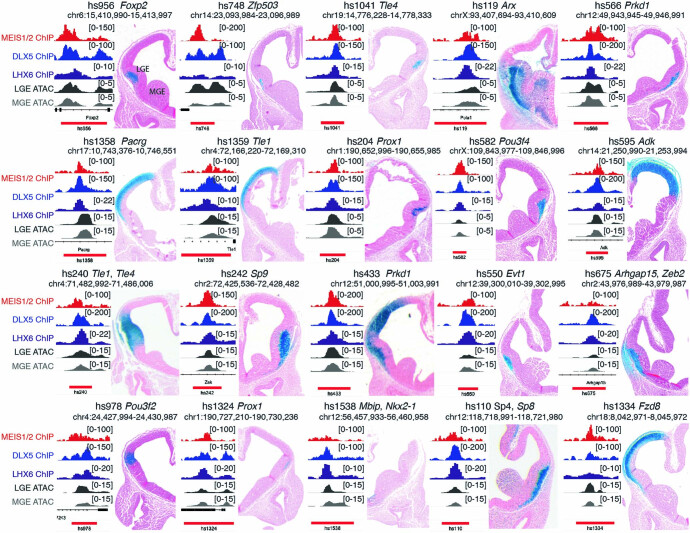
Spatial activity of select enhancers in the embryonic forebrain. Selected Vista enhancers with in vivo activity at E11.5 (data: Visel et al. (2007)) and cobinding of MEIS-DLX5-LHX6. On the left side of each image are panels with representative tracks of GE ChIP–seq of MEIS1/2 at E14.5 (red), DLX5 at E13.5 (blue) (data: Lindtner et al. (2019)), LHX6 at E13.5 (purple) (data: Sandberg et al. (2016)) and scATAC-seq from the LGE (dark gray) and MGE (gray) at E12.5 (data: Rhodes et al. (2022)). MGE, medial ganglionic eminence; LGE, lateral ganglionic eminence.

We intersected ChIP–seq peaks in the GE of MEIS1/2, DLX5 (ref. ^4^) and LHX6 (ref. ^35^). Out of 151 MEIS1/2–DLX5–LHX6 overlapping peaks, 41 were within VISTA enhancers and 28 of these enhancers showed activity in the developing forebrain (Extended Data Figs. 4f,g and 8). We selected three of them to perform reporter assays (Fig. 5b–g and Extended Data Fig. 4h–j): (1) *hs1041*, an enhancer of *Tle4*, which encodes transcription corepressor 4, (2) *hs956*, an enhancer of *Foxp2* and (3) *hs748*, an enhancer of *Zfp503*, which encodes the zinc finger protein TF 503. Genes regulated by the selected enhancers are known to play a role in striatal development^28,36,37^, were expressed in PN precursors and were reduced in several clusters in the gMeis2 tCROP-seq experiments (Source Data for Fig. 1). Consistent with the above findings, MEIS2 potentiated the DLX5-mediated activation of *hs1041*, *hs956* and *hs748* reporters. LHX6 alone had little to no effect on the activation of these enhancers. However, co-expression of LHX6 with MEIS2 and DLX5, resulted in a strong suppression of enhancer activity in all three cases (Fig. 5f,g and Extended Data Fig. 4j). This suggests that LHX6, whose expression is spatially restricted to the MGE, suppresses the DLX5–MEIS2-induced enhancer activation in the MGE. To gather further evidence for this mechanism, we screened 20 VISTA enhancers with overlapping ChIP–seq peaks for LHX6, MEIS1/2 and DLX5 (Extended Data Fig. 8). As expected, none of them exhibited robust activity in the mantle zone of the MGE.

Next, we explored the putative enhancer of *Meis2*, *enhMeis2* (ref. ^20^), which also contained MEIS1/2–DLX5–LHX6 cobinding sites. MEIS2 strongly potentiated the DLX5-mediated activation of *enhMeis2* (Fig. 5h,i), suggesting that in the presence of DLX5, MEIS2 can promote its expression via the activation of *enhMeis2*. LHX6 strongly repressed the MEIS2–DLX5-mediated activation of *enhMeis2*, suggesting that LHX6 suppresses the expression of *Meis2*, consistent with a recent *Lhx6* knockout study in mice^38^. Taken together, this suggests that LHX6 suppresses both the gene expression and function of *Meis2* in the MGE (Fig. 5j and Extended Data Fig. 7).

### *Meis2* and *Lhx6* depletion shifts PN and IN gene modules

**Fig. 6:**
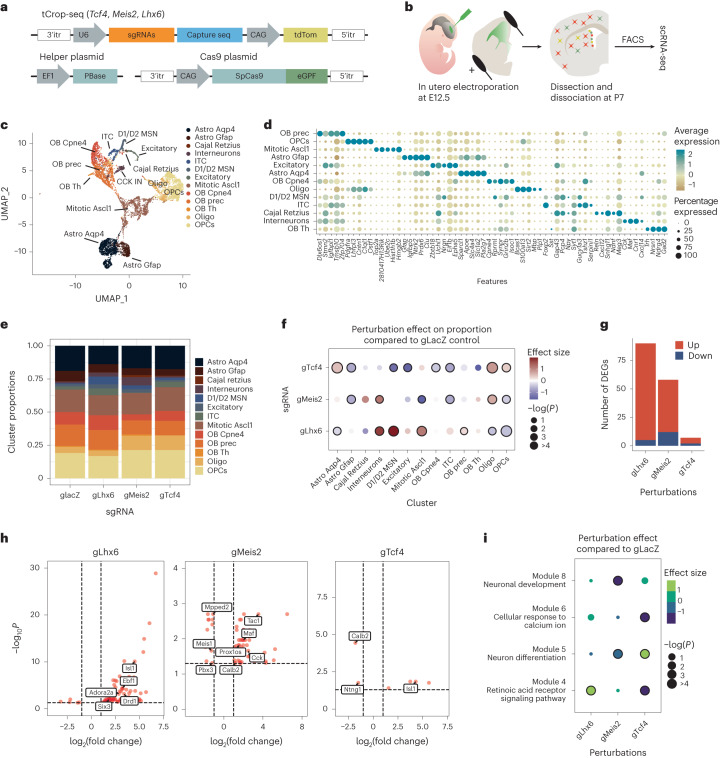
Embryonic disruption of developmental TFs alters postnatal cell types. **a**,**b**, Schematics of tCROP-seq vector maps (**a**) and the experimental workflow (**b**). **c**, UMAP plot of the P7 data colored by cell type (*n* = 8,486 cells). **d**, Dotplot showing the top five marker genes of each cell type. OB, olfactory bulb; prec, precursors; Th, tyrosine hydroxylase expressing cells; OPC, oligodendrocyte progenitor cells; ITC, intercalated cells; MSN, medium spiny neurons; Oligo, oligodendrocyte; Astro, astrocytes. **e**, Cell-type compositions for each sgRNA. **f**, Perturbation effects in different clusters compared to gLacZ controls. Dot color corresponds to effect size, dot size corresponds to −log_10_(*P*). *P* values were derived from Poisson regression models. FDR correction was applied to the *P* values. The black outline indicates statistical significance (adjusted *P* ≤ 0.05). **g**, Bar graph showing the number of DEGs detected in inhibitory neurons for each sgRNA. **h**, Volcano plot showing DEGs in inhibitory neurons for each sgRNA, compared to gLacZ (Methods). The *x* axis represents log_2_(fold change), the *y* axis represents the FDR adjusted −log_10_(*P*). The dotted lines depict a cutoff (adjusted *P* ≤ 0.05, log_2_(fold change) <−1 and >1). **i**, Dotplot showing the effect of perturbation by sgRNAs on the module scores of inhibitory modules. The *P* values were adjusted using Bonferroni correction. A circle denotes statistical significance (−log_2_(*P*) > 3). Source data

**Extended Data Fig. 9:**
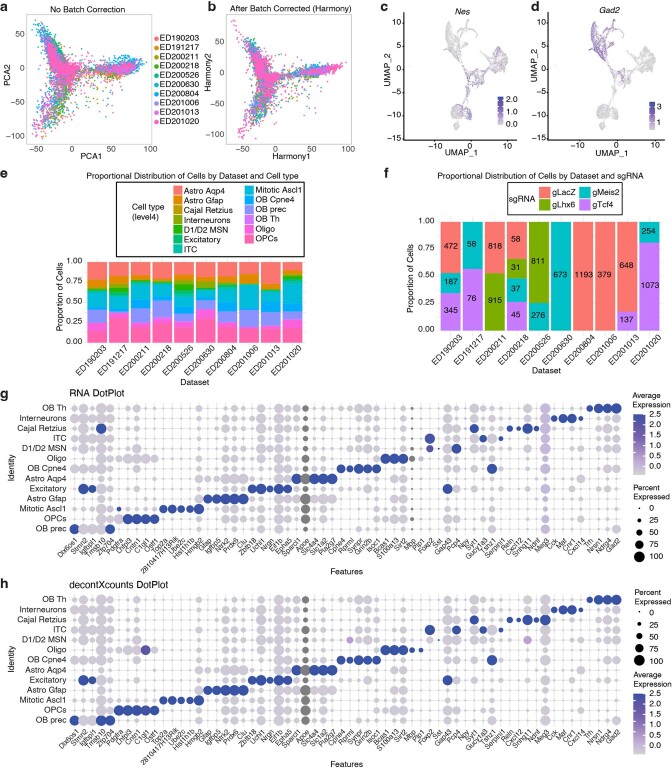
Batch correction and sgRNA coverage of P7 tCROP-seq datasets. **a-b**, 2D visualization of the P7 tCROP-seq dataset pre (**a**) and post (**b**) batch correction using Harmony. **c-d**, Feature plots of canonical marker genes *Gad2* and *Nes* at P7 (n = 8486 cells). **e**, Proportional distribution of cells categorized by dataset and cell type for the P7 tCROP-seq dataset. **f**, Proportional distribution of cells categorized by dataset and sgRNA for the P7 tCROP-seq dataset. **g**, Dotplot showing the top marker genes of inhibitory clusters using the “RNA count" data. **h**, Dotplot illustrating the top marker genes of inhibitory clusters using the‘decontXcounts’ data.

To explore how the depletion of embryonic TFs alter postnatal cell-type composition and identity, we performed pooled tCROP-seq experiments with sgRNAs for *Meis2* (gMeis2), *Lhx6* (gLhx6), *Tcf4* (gTcf4) and LacZ (gLacZ, control). Similar to *Lhx6*, *Tcf4* is a strong predictor of IN fate according to our regression analysis (Fig. 1a), although it is expressed in all GEs^39^ (Extended Data Fig. 7). We delivered sgRNAs via in utero electroporation at E12.5, dissected 35 pups around P7 and performed pooled scRNA-seq. A total of ten scRNA-seq datasets were combined in silico, clustered and annotated on the basis of known marker genes (Fig. 6a–d and Extended Data Fig. 9). Overall, the cell numbers in our postnatal tCROP-seq experiments were lower than in the embryonic datasets, which is largely due to the more difficult dissociation process. To assess the effects of perturbations, we used the methods described by ref. ^40^. All three perturbations had a significant effect on the composition of cell types compared to the gLacZ control (Fig. 6e,f). Cells expressing gLhx6 showed an increased proportion of medium spiny PNs (D1/D2 MSNs), olfactory bulb precursors and INs compared to gLacZ. In addition, consistent with our embryonic tCROP-seq data, the proportion of INs was also increased in gMeis2 compared to gLacZ controls at P7. Cells expressing gMeis2 showed a reduced proportion of intercalated cells of the amygdala (intercalated cells), as well as olfactory bulb inhibitory neurons and oligodendrocyte progenitors (Fig. 6e,f). The gTcf4 expression had a more modest effect on cell proportions, showing only a slight reduction in inhibitory neurons in the olfactory bulb. Each of the gRNAs resulted in DEGs within inhibitory neuron clusters, with many being specific marker genes for either INs or PNs (Fig. 6g,h and Source Data for Fig. 6). The gLhx6 perturbed cells were enriched for projection-neuron-specific genes (*Isl1*, *Foxp1*, *Ebf1*, *Adora2a*, *Drd1* and *Six3*). In contrast, gMeis2 DEGs were enriched for IN-specific genes (*Maf* and *Prox1os*) and depleted for projection-neuron-specific genes (*Mpped2* and *Pbx3*) (Fig. 6h). Our data confirm the in silico prediction: MEIS2 primarily induces PN fate and LHX6 represses it (Fig. 1a).

**Extended Data Fig. 10:**
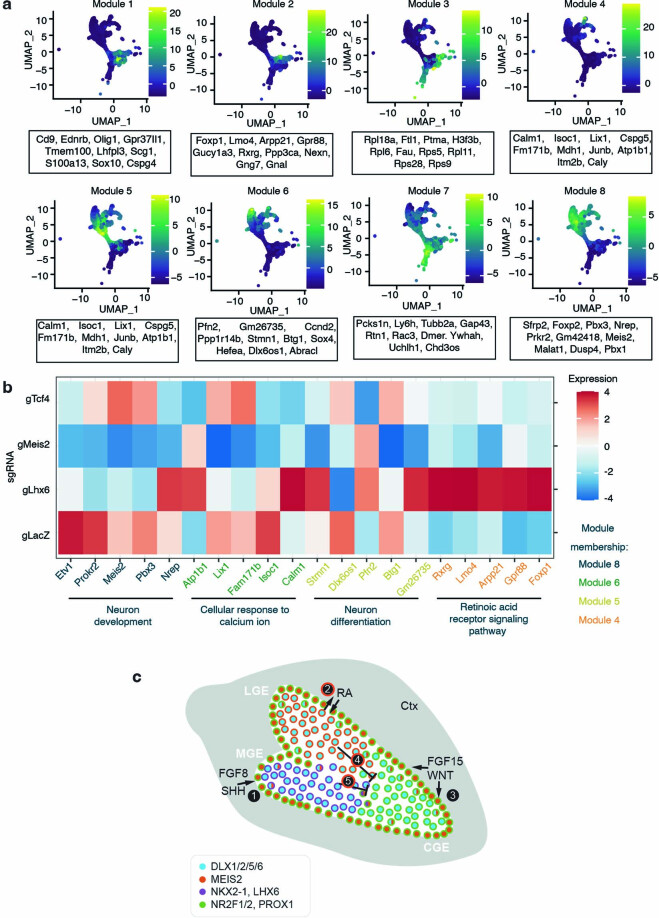
Module analysis of the P7 tCROP-seq dataset. **a**, Feature plots of gene module expression scores and the correlated genes within each module. **b**, Average expression of the top 5 module genes for each sgRNA at P7. **c**, Schematic summary of spatial factors in the ganglionic eminence leading to specific enhancer activation. MGE, medial ganglionic eminence; CGE, caudal ganglionic eminence; LGE, lateral ganglionic eminence; Ctx, cortex; RA, retinoic acid; SHH, sonic hedgehog; FGF, fibroblast growth factor. 1. Storm et al. (2006); Marklund et al. (2004); 2, Molotkova et al. (2007); Chatzi et al. (2011); 3, Borello et al. (2008); Hunt et al. (2023); 4, Su et al. (2022); 5, Vogt et al. (2014); Asgarian et al. (2022). The red circle outline represents the findings of this study.

Next, we performed module analysis using Hotspot^41^, a tool for identifying covarying gene groups. We identified eight gene modules, with four being neuronal (Fig. 6i and Extended Data Fig. 10a,b). Module 5 represented mostly olfactory bulb neuroblasts and contained genes enriched for neuronal differentiation. Module 4 contained medium spiny neuron (MSN) marker genes (for example, *Foxp1*) and genes involved in retinoic acid receptor signaling (*Rarb* and *Rxrg*). The retinoic acid pathway is involved in the switch between proliferation and differentiation^42^ and is essential for striatal development^30,31^. Module 8 contained *Meis2*, as well as some of its target genes, such as *Pbx3* and *Etv1* (Source Data for Fig. 3). Module 6 contained genes involved in calcium response and synapse organization. The perturbation of *Lhx6* was positively associated with the expression of module 4. The perturbation of *Meis2* lowered the expression of both modules 8 and 5. The perturbation of *Tcf4* had a significant effect across modules 6, 5 and 4, consistent with previous findings showing that TCF4 is a key facilitator of neurogenesis and neuronal differentiation^43^ (Fig. 6i). Taken together, the tCROP-seq data at P7 indicate a marked influence of MEIS2, LHX6 and TCF4 on inhibitory neuron specification.

## Discussion

In this study, we explored the role of the TF MEIS2 in the development of GABAergic PNs and INs in the murine telencephalon. The study used a method that combines transposon-based strategies for CRISPR perturbation sequencing (tCROP-seq) and barcode lineage tracing (TrackerSeq). Using transposon-based strategies, we not only improved the efficiency of CRISPR–Cas perturbation sequencing compared to lentivirus-based approaches but also enabled the combination of CRISPR–Cas perturbation with barcode lineage tracing. Consistent with a previous study in which a conditional *Meis2* knockout mouse line was used^5^, CRISPR–Cas perturbation of *Meis2* decreased the expression of projection-neuron-specific genes and reduced the generation of LGE-like GABAergic PN types. Moreover, CRISPR–Cas perturbation of *Meis2* increased the proportion of IN types and shifted the clonal production of postmitotic precursors in the GE from LGE-like PN precursors to CGE- and MGE-like IN precursors. We conducted a MEIS1/2 ChIP–seq and in vitro reporter assays and found that MEIS2 requires the presence of DLX proteins to direct its functional activity toward regulatory enhancers of projection-neuron-specific genes containing specific DLX–MEIS cobinding sites.

Our findings contribute to an overall picture in which spatial selective enhancer activation plays a role in the early acquisition of GABAergic identities (Extended Data Fig. 10c). Different GABAergic cell types arise from regional differences in the specification of GE progenitors, which are initially established by morphogenic molecules such as retinoic acid (LGE)^29,31^, fibroblast growth factor (FGF)8 and sonic hedgehog (SHH, MGE)^44,45^, FGF15 and WNT (CGE)^46,47^ and their downstream TFs, such as MEIS2 (LGE), NKX2.1 and LHX6 (MGE) and NR2F1/2 (CGE). The tissue specificity of members of the DLX family in the GE directs the functional activity of MEIS2 to regulatory sites related to GABAergic PN development. TALE TFs (for example, MEIS) have previously been shown to act as broad co-activators of homeobox genes^48^ and several studies have demonstrated that MEIS proteins require the presence of other TFs, such as PBX, HOX, TBX and PAX6, to promote differentiation in the limbs, heart, lens, hindbrain and olfactory bulb^48–52^.

We showed that in the GE, MEIS2 and DLX5 together activate several enhancers associated with PN gene expression that are active in the LGE. This spatial component appears to be partially mediated by LHX6, which antagonizes MEIS2 in two ways as follows. First, we showed that LHX6 suppresses an enhancer of *Meis2*, probably resulting in repression of *Meis2* gene expression in the SVZ/mantle zone of the MGE. In line with this, both the conditional knockouts of *Lhx6* and *Nkx2-1*, which act upstream of LHX6, resulted in increased expression of *Meis2* (refs. ^35,38^). Second, we showed that LHX6 can efficiently repress the MEIS2–DLX5-induced activation of PN enhancers. This could represent a mechanism to suppress the activation of PN enhancers within cells at the transition between the VZ and SVZ of the MGE. In this area, we identified cells co-immunolabeled for MEIS2 and LHX6, potentially attributed to the time required for MEIS2 protein degradation. The suppression by LHX6 could be mediated by a competition of LHX6 with DLXs for the common DNA-binding motif TAATT^4,35^. Alternatively, LHX6 could restrict the interaction of MEIS2–DLX5 with DNA through direct binding to DLX5 or MEIS2. LHX6 belongs to the LIM domain homeodomain (LIM-HD) protein family, which is characterized by two cysteine-rich LIM domains for protein–protein interactions and a homeodomain for binding DNA^53^. For example, LHX6 directly interacts with PITX2 to inhibit its transcriptional activities^54^. In parallel, other transcriptional programs are probably involved in the repression and activation of PN and IN cell fate^2,55^. While our study focuses on the MGE, it is worth noting that in the CGE, MEIS2 expression and function may be subject to regulation by other factors, such as PROX1, NR2F1/2 or SP8. Notably, NR2F1/2 and SP8 exhibit a caudal–rostral expression gradient in the forebrain, which appears to be in contrast to the expression pattern of MEIS2 (ref. ^56^), suggesting a potential interplay in gene expression regulation. In the gLhx6 tCROP-seq experiment, we observed an elevated proportion of LGE-like PNs, which has not been reported in a study of *Lhx6* knockout mice^33^. However, this study found a periventricular ectopy in a substantial cell population, which has not been investigated further. These cells might correspond to the increased PN cell group we observed. In our postnatal tCROP-seq dataset, the recovered numbers of INs and PNs are limited, impeding the distinction between MGE and CGE INs and preventing fine-grained subtype analysis.

Others^57^ propose two *cis*-regulatory strategies which could drive cell fate choice in developing neural progenitors. One—differential binding—relies on a common regulatory landscape, whereby the different composition of TFs at these CREs dictates differential gene expression and cell fate decisions. The other—differential accessibility—relies on cell-type-specific chromatin remodeling. Our results support the first strategy. While the selected enhancers in our study were accessible throughout all GEs, our data show that their activity depends on the TFs composition. For example, the *Foxp2* enhancer *hs956* is not active in the ventricular zone of the GE, probably because *Dlx* genes are absent in the ventricular zone. This enhancer is active in the SVZ and mantle zone of the LGE, where both *Meis2* and *Dlx* genes are expressed. The enhancer is not active in the SVZ/mantle zone of the MGE where *Meis2* is absent and a repressive TF such as *Lhx6* is present.

How do MEIS2 and DLX5 work together? Others^52^ performed pull-down experiments with a tagged form of MEIS2 using olfactory bulb tissue and detected DLX-specific protein bands in the MEIS2 precipitates. This could either indicate a direct protein interaction or be the result of a process called ‘DNA-guided cooperativity’, a mechanism where certain TFs cooperatively bind to adjacent DNA sites without forming stable, direct protein–protein interactions^58^. This form of cobinding is guided by the DNA sequence itself, rather than by protein–protein interactions. Support for DNA-guided cooperativity as the mechanism underlying the interaction between MEIS and DLX comes from a study by ref. ^25^, who performed in vitro analyses of TF pairs, including a crystal structure of MEIS1 and DLX3 bound to their identified recognition site. Their results suggested that the interactions between MEIS and DLX are predominantly mediated by DNA.

Haplo-insufficiency of the MEIS2 in humans results in an autosomal dominant disease characterized by several congenital malformations, mild-to-severe intellectual disability with poor speech and delayed psychomotor development^6,7^. The amino acid Arg333, located in the homeodomain of MEIS2, is highly conserved across species and isoforms and was found mutated in several patients with severe disease. Our study found that the missense mutation p.Arg333Lys led to a strong decrease in enhancer activation compared to normal MEIS2. Owing to the location of Arg333 in the homeodomain of MEIS2, it is likely that the mutations in this amino acid interfere with the DNA-binding ability of the protein. This could result in a change in GABAergic cell-type proportions, in particular a reduced number of PNs in the striatum, caused by disturbed fate decisions during embryogenesis.

The efficiency with which MEIS2 can co-activate selective enhancers suggests a general strategy for implementing spatial information to generate distinct cellular populations. The ability of MEIS2 to induce context-specific cell types may exemplify how certain subsets of cells in different parts of the body are affected in developmental disorders. Further research is needed to fully comprehend the intricate interactions between TFs and cofactors in the regulation of cell fate decisions during GABAergic neuron development and their potential implications in human disease.

## Methods

### Mice and in utero surgeries

All experiments were conducted according to institutional guidelines of the Max Planck Society and the regulations of the local government ethical committee (Beratende Ethikkommission nach §15 Tierschutzgesetz, Regierung von Oberbayern). All mouse colonies were maintained in accordance with protocols approved by the Bavarian government at the Max Planck Institute for Biological Intelligence or the Helmholtz Zentrum in Munich. Mice at the Max Planck Institute were group housed in isolated ventilated cages (room temperature 22 ± 1 °C, relative humidity 55% ± 5%) under a 12 h dark/light cycle with ad libitum access to food and water. C57BL/6NRj wild-type females (from inhouse breeding) were crossed to C57BL/6NRj wild-type or to CAS9-EGFP (B6.Gt(ROSA)26Sortm1.1(CAG-cas9*,-EGFP)Fezh/J, JAX, 026179) males^10^. Embryos were staged in days post coitus, with E0.5 defined as 12:00 of a day that a vaginal plug was detected after overnight mating. Timed pregnant mice were anesthetized with isoflurane (5% induction, 2.5% during the surgery) and treated with the analgesic Metamizol (WDT). A microsyringe pump (Nanoject III Programmable Nano-liter Injector, DRUM3-000-207) was used to inject ~700 nl of DNA plasmid solution made of 0.6 μg μl^−1^ of pEF1a-pBase (*piggyBac* transposase; a gift from R. Platt) and the sgRNA plasmid 0.7 μg μl^−1^, diluted in endo-free TE buffer and 0.002% Fast Green FCF (Sigma, F7252), into the lateral ventricle. pCAG-Cas9-EGFP (a gift from R. Platt) plasmid was added when wild-type males were used for plugs. For TrackerSeq experiments, a barcode library (final concentration of 0.4 μg μl^−1^) was added to the DNA plasmid solution. Embryos were then electroporated by holding the head between platinum-plated tweezer electrodes (5 mm in diameter, BTX, 45-0489) across the uterine wall, while five electric pulses (35 V, 50 ms at 1 Hz) were delivered with a square-wave electroporator (BTX, ECM830)^59^. We used these relatively large electrodes to target all areas of the GE (MGE, CGE and LGE)^60^. Pups were kept with their mothers. To assess cellular distribution after in utero electroporation, embryos were collected at E16.5 and E18.5. Dissected brains were fixed overnight in 4% paraformaldehyde (Electron Microscopy Sciences, 15710) and washed with PBS. The 50 μm tissue sections were prepared on a Leica VT1200S Vibratome and mounted on slides with ProLong Glass Antifade Mountant (P36980, ThermoFisher). All images were acquired using a STELLARIS 5 confocal microscope system (Leica). For immunohistochemistry, C57BL/6 wild-type brains were prepared from three E13.5 embryos, postfixed in 4% PFA solution for 2.5 h and subsequently washed with PBS.

### TrackerSeq library preparation and validation

TrackerSeq is a *piggyBac* transposon-based^61^ lineage tracing tool that is compatible with the 10x Genomics Chromium platform^8^. It records clonal lineages of single cells through the integration of oligonucleotide sequences into the genome of mitotic progenitors. Each lineage barcode is a 37 bp long synthetic nucleotide that consists of short random nucleotides bridged by fixed nucleotides. We followed the protocols from ref. ^8^ to prepare TrackerSeq plasmids. Briefly, an oligo library was cloned downstream of the Read2 partial primer sequence in the purified donor plasmid via Gibson Assembly reactions (NEB, E2611S). Gibson assembly reactions were then pooled and desalted with 0.025 μm MCE membrane (Millipore, VSWP02500) for 40 min and concentrated using a SpeedVac. A total of 3 μl of the purified assembly was incubated with 50 μl of NEB 10-*β*-competent *Escherichia coli* cells (NEB, C3019H) for 30 min at 4 °C, then electroporated at 2.0 kV, 200 Ω, 25 μF (Bio-Rad, Gene Pulser Xcell Electroporation Systems). Electroporated *E. coli* were incubated for 90 min shaking at 37 °C and plated on prewarmed sucrose/ampicillin plates. The colonies were scraped off the plates 8 h later and the plasmids were grown in LB medium with ampicillin up to optical density 0.5. The plasmid library was purified using a column purification kit (Zymo, D4202). We first assessed the integrity of the TrackerSeq barcode library by sequencing it to a depth of ~42 million reads to test whether any barcode was over-represented. Around 3.6 million valid lineage barcodes which had a quality score of 30 or higher were extracted from the R2 FASTQ files using Bartender^62^. One-thousand barcodes were randomly sampled from the extracted lineage barcodes to assess hamming distance. To group similar barcodes into putative barcodes, Bartender assigns a UMI to each barcode read to handle polymerase chain reaction jackpotting errors and clusters them. The cluster distance was set to 3. A total of 2 × 10^5^ clusters of barcodes were identified.

### Immunostainings

Paraformaldehyde-fixed brains at E13.5 and E18.5 were incubated in 10%, 20% and 30% sucrose for 24 h each, embedded in Neg-50 Frozen Section Medium (Epredia, 22110617) and subsequently snap-frozen in isobutane at −70 °C. The 16 μm tissue sections were prepared on a Thermo Scientific CryoStar NX70 Cryostat and transferred to glass slides. Sections were incubated overnight with primary antibodies anti-MEIS2 (SCBT, sc-515470-AF594, 1:250), anti-LHX6 (SCBT, sc-271433-AF488, 1:50), anti-PROX1 (R&D Systems, AF2727, 1:250) and anti-CTIP2 (Abcam, ab18465, 1:500). Sections were then incubated with secondary antibodies at room temperature for 2 h at 1:500 dilution: anti-rabbit AF594 (Invitrogen, A21207); anti-rat AF488 (Invitrogen, A21208); and anti-goat AF488 (Invitrogen, A11055). Nuclei were counterstained with DAPI and slides mounted with Aqua-Poly/Mount (Polysciences, 18606). Fluorescence imaging was conducted on a LSM880 confocal microscope (Zeiss Microscopy) using Plan-Apochromat 20/0.8 M27 or C-Aprochromat 63×/1.2 W Korr M27 objectives.

### Sample collection

Before preparing brain tissue for scRNA-seq, each brain was examined under a stereo microscope and only brains that met the following criteria were selected for scRNA-seq:

1. Dispersed tdTomato+ neurons throughout the neocortex. This indicates that we targeted MGE/CGE-derived INs which migrate long distances and disperse to different cortical brain regions.
2. Dense tdTomato+ neurons throughout the striatum. MSNs are known to originate from the LGE and account for ~90% of the neurons in the striatum.
3. tdTomato+ neurons in the olfactory bulb. GABAergic precursors are known to migrate from the LGE to the olfactory bulb.

We performed immunohistochemical labeling to validate that after in utero electroporation, individual brains express sgRNAs in cortical INs derived from the MGE (anti-SST) and CGE (anti-PROX1), as well as in striatal MSNs derived from the LGE (anti-CTIP2). We collected electroporated brains from mouse embryos (both sexes) at E16.5 in ice-cold Leibovitz L-15 Medium (ThermoFisher, 11415064) with 5% FBS or at P7–8 in ice-cold Hibernate-A Medium (ThermoFisher, A1247501) with 10% FBS and B-27 supplement (ThermoFisher, 17504044). Forebrain tissue was manually dissected. A papain dissociation system (Wortington, LK003150) was used according to the protocol described in ref. ^40^ on the gentleMACS Octo Dissociator (Miltenyi Biotec) to generate a cell suspension. To isolate positive cells, flow cytometry was performed using a BD FACSAria III Cell Sorter (BD FACSDiva Software, v.8.0.2) with a 100 μm nozzle. EGFP and tdTomato+ cells were collected in bulk to test sgRNA *Meis2* knockout efficiency following the in vitro protocol (above; results in Supplementary Table 1) or for downstream processing on the 10x Genomics Chromium platform. After sorting in PBS (Lonza, 17-516) with 0.02% BSA (B9000, NEB), 5,000–16,000 individual cells per sample were loaded onto a 10X Genomics Chromium platform for gel beads-in-emulsion and complementary DNA generation, carrying cell- and transcript-specific barcodes using the Chromium Single Cell 3' Reagent Kit v.3.1 with Feature Barcoding technology (10X Genomics, PN-1000121) following the manufacturer’s protocol (document no. CG000205, 10X Genomics).

### tCROP-seq

To investigate the effects of TF perturbation on cellular fate decisions in a sparse population of precursors in the GE, we modified CROP-seq^9^, a method that enables CRISPR–Cas perturbation with scRNA-seq readout. Instead of lentiviral vectors, we applied a *piggyBac* transposon-based strategy (tCROP-seq) and in utero electroporation to deliver sgRNAs to cycling progenitors in the GE (Fig. 1b). The transposon system allows genes to be stably integrated into the genomes of electroporated cells and thus to be transmitted to their postmitotic daughter cells^61^. This increases the pool of perturbed cells and ensures that the perturbation occurs during a period covering the peak of neurogenesis^8^. We also added specific capture sequences to the sgRNA vectors which efficiently link sgRNAs to cell barcodes and enable sequencing of the protospacer from the transcriptome^63^. The tCROP-seq sgRNA vectors also encode tdTomato to enable the labeling and enrichment of perturbed neurons. The efficiency of sgRNA *Meis2* to induce frame-shift mutations was validated in vitro and in vivo before the tCROP-seq experiments (Supplementary Table 1).

### Preparation of tCROP-seq libraries

We used the Feature Barcode technology from 10X Genomics to prepare tCROP-seq libraries. The assay captures transcriptomes and guide RNAs from the same cell. We generated 3' gene expression and gRNA libraries according to the manufacturer’s manual (document no. CG000205) using the Chromium Library v.3.1 kit (PN-1000121), Feature Barcode Library Kit (PN-1000079) and Single Index Kit (PN-1000213) from 10X Genomics. The quantification of the libraries was performed with an Agilent BioAnalyzer.

### Preparation of TrackerSeq NGS libraries

The TrackerSeq lineage libraries were amplified from 10X Genomics cDNA libraries with the Q5 polymerase (NEB, M094S) in a 50 μl reaction, using 10 μl of cDNA as template^8^. Specifically, each PCR contained: 25 μl of Q5 High-fidelity 2X Master Mix, 2.5 μl of 10 μM P7-indexed reverse primer, 2.5 μl of 10 μM i5-indexed forward primer, 10 μl of molecular grade H_2_O, 10 μl of cDNA (for primer sequences and indices, see Supplementary Table 4). Libraries were purified with a dual-sided selection using SPRIselect (Beckman Coulter, B23318) and quantified with an Agilent BioAnalyzer.

### Sequencing and read mapping

We sequenced the transcriptome and CRISPR barcode libraries using an Illumina NextSeq 500 at the Next-Generation Sequencing Facility of the Max Planck Institute of Biochemistry or a NovaSeq at the Genomics Core Facility at the Helmholtz Center in Munich. Full details on each dataset are provided in Supplementary Table 2. The sequencing reads in FASTQ files were aligned to a reference transcriptome (mm10-2.1.0) and converted into UMI counts using the 10X Genomics Cell Ranger software (v.3.0.2 or 5.0.1).

### tCROP-seq preprocessing

We loaded the UMI count data into R and processed it using the Seurat (v.4) package^13^. To recover the CRISPR gRNAs, we used Cell Ranger^64^, which produced a CSV file listing the cell barcodes and the sgRNA detected for each cell.

#### Processing embryonic tCROP-seq datasets

Electroporation of ventral progenitors using the 5 mm electrode targets additional progenitors located adjacent to the GE. These include progenitors of excitatory neurons located at the border between the pallium and the subpallium. Thus, our dataset consisted of: inhibitory, 16,098 neurons; excitatory, 10,010 neurons; glial, 5,915 cells; pericytes, 1,008 cells; fibroblasts, 537 cells; macrophages, 523 cells; and blood, 390 cells. We focused only on cells from inhibitory clusters where a gRNA could be recovered and excluded the others. We integrated inhibitory neurons with scRNA-seq datasets from wild-type mice^8^ to get a higher resolution of inhibitory cell states (Fig. 1) using the integration tool from Seurat^13^. We obtained cluster-specific marker genes by performing differential expression analysis (see below). Clusters were assigned to cell types on the basis of the expression of known marker genes, primarily using http://mousebrain.org/development/ (ref. ^65^) and https://DropViz.org (ref. ^66^).

#### Processing postnatal tCROP-seq datasets

To process the P7 datasets, we integrated Harmony (v.1.0)^12^ into our Seurat^13^ workflow for batch correction, using default settings (theta = 2, lambda = 1, sigma = 0.1). We used the first 30 Harmony embeddings for uniform manifold approximation and projection (UMAP) visualizations and clustering analysis. To group cells into clusters, we first constructed a shared-nearest neighbor graph from Harmony embeddings using the FindNeighbors() algorithm, then input the graph into the FindClusters() function in Seurat (dimensions = 30, res = 0.8). To test whether our postnatal dataset was subject to non-specific background expression, we applied DecontX^67^ using the default parameters. We retrieved the count matrix from our Seurat object, created an SCE object, ran DecontX and then added the corrected count matrix back to the Seurat object. The difference before and after correction was relatively small. Therefore, we decided to use the uncorrected counts for the subsequent analysis.

### Logistic regression model to predict IN and PN genes

We used a recently published scRNA-seq dataset from ref. ^8^ to explore genes that are predictive for IN or PN fate. Raw counts for samples from GE-specific microdissections collected from wild-type mice at E13.5 and E15.5 were processed using Seurat (v.4.1.0)^13^. After integration across batches, counts were normalized and scaled. Cluster annotations from ref. ^8^ were summarized into four broad cell classes: mitotic, trunk, IN and PN. For performing logistic regression, we subsetted cells from IN and PN cell classes. Logistic regression was performed using the 3,000 most variable genes. To account for balanced design, cells were subsampled to have a equal number of cells in both classes. A logistic regression model was trained on the scaled expression matrix of the corresponding cells and genes, where two-thirds of cells were used for training and the other third for validation. This was implemented using the cv.glmnet(family = ”binomial”) function from the R package glmnet^68^. The model achieved 99.15% accuracy on the held-out validation set. For each gene, the model predicts a coefficient which reflects whether high expression of the gene is predictive of a cell being an IN (coefficient ∈ [0,0.5]) or a PN (coefficient ∈ [0.5,1]).

### Comparing cell-type composition between perturbations

We compared the perturbation effect on cell-type composition using the method described by ref. ^40^. A script of the analysis is deposited on GitHub (https://github.com/mayer-lab/Dvoretskova-et-al). Compositional change was investigated using the CellComp_Poisson R function from ref. ^40^. It performs Poisson regression analysis to identify genes that are differentially expressed across different cell types, perturbations and batches. First, the function performs data cleaning by creating a metadata data frame and filtering out cells with low counts. It then fits a Poisson regression model for each combination of cell type and perturbation and extracts the coefficients for the perturbation variable. These coefficients are then used to calculate *P* values and adjusted *P* values for each gene.

### Differential gene expression analysis

We used the Libra package (v.1.0) to perform differential gene expression analysis^69^. We ran the run_DE function on Seurat objects using the following parameters: de_family = pseudobulk, de_family = pseudobulk, de_method = edgeR, de_type = LRT. We obtained DEGs of PNs or INs by using the run_DE function on cells grouped into classes (mitotic, PNs and IN). We filtered for statistically significant genes (false discovery rate (FDR)-adjusted *P* value threshold = 0.05). Genes were considered differentially expressed if log_2_(fold change) <−0.3 and >0.3 for embryonic and log_2_(fold change) <−1 and >1 for postnatal datasets.

We also used the R packagage Libra to calculate the DEGs for each cluster (i_Calb2/Nxph1, i_Cck/Reln, i_Ebf1/Zfp503, i_Foxp1/Isl1, i_Foxp1/Six3, i_Isl1/Bcl11b, i_Lhx6/Npy, i_Meis2/Bcl11b, i_Nfib/Tcf4, i_Nr2f2/Nnat, i_Tiam2/Zfp704 and i_Tshz1/Pbx1). The result of the DEG analysis is in the Source Data of Fig. 3. We applied thresholds (adjusted *P* ≤ 0.05 and log_2_(fold change) <−1.0 and >1.0) to select the genes for intersection with the ChIP–seq data. For the Venn diagram, we combined DEGs from all subtypes and split them into upregulated or downregulated genes.

### TrackerSeq (lineage tracing) barcode processing and analysis

For a subset of datasets (ED210204, ED210215, ED211111 and ED211124), we included TrackerSeq lineage barcodes to perform a clonal analysis. We followed the protocol outlined in ref. ^8^ to process the TrackerSeq barcodes to obtain cloneIDs for each corresponding cell barcode. The resulting cloneIDs were added to the Seurat object metadata. To quantify clonal relationships between cell classes, the inhibitory clusters were first merged into cell classes (Fig. 2c) on the basis of whether they were annotated as mitotic (*Ube2c* and *Top2a*) or as INs and PNs (*Gad2*). The UpsetR library was used to count the number of clones shared between the neuronal classes, as well as the proportion of clonal relationships in gMeis2 and gLacZ datasets. The set size is the number of cells in the class. The Upset bar plot shows the calculated proportion of each type of clonal distribution category within the perturbation. The calculated percentage stemmed from dividing the number of clones in a given category (for example, clones containing only mitotic cells and IN) by the total count of clones spread across all clonal distribution categories.

To assess clonal coupling, we used a method from ref. ^70^. The method computes an observed/expected ratio of shared barcodes for each pair of cell states. A barcode is considered shared if it appears in at least one cell from both states. From the observed shared barcode matrix *O*_*ij*_, it derives an expected shared barcode matrix *E*_*ij*_ under the assumption of no lineage couplings, as follows:

$${E}_{ij}=\frac{\mathop{\sum}\nolimits_{k}{O}_{kj}\times \mathop{\sum}\nolimits_{k}{O}_{jk}}{\mathop{\sum}\nolimits_{k,l}{O}_{kj}}$$

These matrices were recomputed 1,000 times, each time using a random 25% sample of clones. The lineage coupling scores shown in Extended Data Fig. 2g represent the median *O*_*ij*_/*E*_*ij*_ from these 1,000 randomized trials. To assess significance, we calculated empirical *P* values for each pair of cell states. An observed/expected ratio of 1 indicates lineage coupling that is in line with random expectations, a ratio of <1 or >1 indicates lower or higher lineage coupling, respectively. Empirical *P* values were calculated by counting the number of random shuffles, where the simulated observed/expected ratio was higher than 1 for negatively coupled pairs or lower than 1 for positively coupled pairs of cell states. Empirical *P* values were subsequently corrected for multiple testing using FDR correction.

### Hotspot gene module analysis

Hotspot (v.0.91) is a tool for identifying co-expressing gene modules in a single-cell dataset^41^. It computes gene modules by evaluating the pairwise correlation of genes with high local autocorrelation, then clusters the results into a gene–gene affinity matrix. To identify the inhibitory-specific modules in the postnatal dataset, we first separated the *Gad2*-expressing inhibitory neuron population from the rest of the P7 dataset. We ran the depth-adjusted negative binomial model on the entire count matrix and Harmony (v.1.0) corrected principal components. We computed a *k*-nearest-neighbors graph with 30 neighbors, 9,154 non-varying genes were subsequently detected and removed. Autocorrelations between each gene were calculated and the top 500 significant (FDR ≤ 0.05) genes were used to evaluate pairwise gene associations (local correlations). After pairwise local correlations were calculated, we grouped genes into modules. Modules were created through agglomerative clustering, where the minimum number of genes per module was set to 30. Eight modules were identified and 103 genes were not assigned to a module. Summary per-cell module scores is calculated using the calculate_module_scores() function as described by ref. ^41^. As described by ref. ^40^, linear regression was used to test the relationship between perturbation and Hotspot module gene scores. We fitted a linear regression model that accounted for the batch and number of genes and extracted the effect sizes to estimate how the module scores in the perturbed cells deviated from gLacZ control cells^40^. For the three TFs, the perturbations had significant effects across different modules.

### GO term analysis

Gene ontology (GO) term analysis was done using the package enrichR (v.3.0)^71^. The DEGs and module genes of each module were queried against the following databases: GO_Molecular_Function_2018, GO_Cellular_Component_2018 and GO_Biological_Process_2018. Only GO terms that were significant (adjusted *P* ≤ 0.05) were kept.

### Luciferase assay

CREs were amplified from mouse genomic DNA with the Q5 polymerase (NEB, M0491) using primers listed in Supplementary Table 5 and cloned into pGL4.24[luc2P/minP] (Promega, E8421) with the NEBuilder HiFi DNA Assembly kit (NEB, E2621). The enhancer *hs1080* had to be cloned in reverse-complement. Mouse *Meis2* isoform D (4) (the tag was removed) and *Lhx6* variant 1 (C-DYK) expression vectors were purchased from Genscript. *Dlx5* and *Pbx1* coding sequences were amplified from mouse cDNA and cloned into pcDNA3.1 (Genscript). The *Meis2* vector was mutated with the NEBuilder HiFi DNA Assembly kit (NEB, E2621) to harbor the human mutation p.(Arg333Lys), c.998G>A (Meis2*333)^7^. A short version of the *enhD1* luciferase vector was mutated using gBlock (IDT) and the NEBuilder HiFi DNA Assembly kit. Luciferase reporter vectors were cotransfected with pNL1.1.PGK[Nluc/PGK] (Promega, N1441) and different combinations of pcDNA3, pcDNA3-Dlx5, pcDNA3-PBX1, pcDNA3-Meis2 and pcDNA3-Lhx6. Neuro2a cells were seeded in 24-well plates at 80,000 cells per well and were transfected on the next day with TransIT-LT1 Transfection Reagent (Mirus, MIR 2300), using 150 ng of luciferase reporter, 10 ng of Nluc/PGK and 350 ng of total of pcDNA3.1 plasmids per well (150 ng per TFs vector). pcDNA stands for a control plasmid (pcDNA3.1) which does not contain a protein coding sequence. The pcDNA was used to balance the DNA load during transfections. Cells were harvested 24 h after transfection and luciferases activity was measured using the Nano-Glo Dual-Luciferase Reporter Assay System (Promega, N1630) on a Berthold Multimode reader Tristar2S. A Nanoluc reporter was used for normalization. Statistical tests were performed using the GraphPad Prism software (v.10.0.2). Two-way analysis of variance (ANOVA) followed by Tukey’s honestly significant difference (HSD) test were used to determine the statistical significance between various conditions. Data distribution was assumed to be normal but this was not formally tested. All results for statistical analysis are listed in Source Data files.

### Chromatin immunoprecipitation

Mice were handled in accordance with the CNIC Ethics Committee, Spanish laws and the EU Directive 2010/63/EU for the use of animals in research. GEs and part of the underlying striatum of 70 wild-type C57BL/6 embryos at E14.5 were microdissected and immediately fixed in 1% formaldehyde for 5 min. Tissue preparation, immunoprecipitation and sequencing on an Illumina HiSeq2500 were performed as previously described^50^. Immunoprecipitation was carried out using a combination of two anti-MEIS, one recognizing MEIS1A and MEIS2A, the other recognizing all MEIS2 isoforms^72^.

### ChIP–seq data analysis

Single-end reads of 61 bp length were trimmed using Cutadapt (v.1.16) and mapped to GRCm38 using Bowtie2 (v.2.3.0)^73^ followed by duplicate removal with Picard (v.2.15) and peak calling with MACS2 (v.2.1.2)^74^ using a cutoff of *q* ≤ 0.01. TSS definitions were adapted from the eukaryotic promoter database (mmEPDnew v.003)^75^. We determined the distance of each peak to the nearest TSS using the R package Plyranges (v.1.180). Using custom R scripts, peaks were assigned to the TSS of a gene when overlapping an ~5 kb region around a TSS, defined as promoter region. Overlap with developmental enhancers^20^ was determined in the same way. Similarly, we determined overlap of MEIS2-binding sites with DLX5-binding sites at E13.5 from ref. ^4^ and LHX6-binding sites at E13.5 from ref. ^35^. Enrichment of enhancer-overlapping peaks among shared MEIS2/DLX5 peaks, compared to MEIS2- and DLX5-exclusive peaks, was determined using Pearson’s Chi-squared test of the R stats package (v.4.0.2). Genomic tracks and VISTA enhancers^26^ were visualized using the Integrated Genomics Viewer (v.2.4.1)^76^.

Motif identification and enrichment of known motifs were carried out by HOMER (v.4.10.4)^77^ using default settings. Motif enrichment within enhancer- and promoter-overlapping peaks was likewise performed with HOMER. We used SpaMo (v.5.4.1)^78^ to determine motif spacing between MEIS2 and DLX5-binding motifs in common MEIS2/DLX5-binding sites, within 100 bp upstream and downstream of MEIS2 peak summits.

### Data used in this study

GSE167047 (snATAC-seq of E12.5 MGE and LGE; ref. ^27^), GSE85705 (LHX6 ChIP–seq GE E13.5; ref. ^35^), GSE124936 (DLX1, DLX2 and DLX5 ChIP–seq GE E13.5; ref. ^4^) and GSE188528 (scRNA-seq of LGE, MGE, CGE E13.5; ref. ^8^) were downloaded from https://www.ncbi.nlm.nih.gov/geo. Coordinates of developmental enhancers and interacting genes were taken from ENCODE^20^. VISTA enhancer images were downloaded from the VISTA Enhancer Browser (https://enhancer.lbl.gov)^26^.

### Statistics and reproducibility

Data distribution was assumed to be normal but this was not formally tested. The exact values of *n* indicating the total number of animals per group are reported in each figure caption or in the Source Data files. Analyses were carried out using Prism v.10.0.2, R v.3.6 and R v.4.1. The sample size was chosen empirically or based on preliminary data to provide a sufficient level of statistical power for detecting indicated biological effects. No statistical methods were used to predetermine sample sizes but our sample sizes are similar to those reported in previous publications^8^. No data were excluded from the analyses. The experiments were not randomized.

### Reporting summary

Further information on research design is available in the Nature Portfolio Reporting Summary linked to this article.

## Online content

Any methods, additional references, Nature Portfolio reporting summaries, source data, extended data, supplementary information, acknowledgements, peer review information; details of author contributions and competing interests; and statements of data and code availability are available at 10.1038/s41593-024-01611-9.

### Supplementary information

Supplementary Information Supplementary Tables 1–5.

Reporting Summary

### Source data

Source Data Fig. 1 Statistical source data.

Source Data Fig. 2 Statistical source data.

Source Data Fig. 3 Statistical source data.

Source Data Fig. 4 Statistical source data.

Source Data Fig. 5 Statistical source data.

Source Data Fig. 6 Statistical source data.

Source Data Extended Data Fig. 2 Statistical source data.

Source Data Extended Data Fig. 3 Source data.

Source Data Extended Data Fig. 4 Statistical source data.

Source Data Extended Data Fig. 5 Statistical source data.

Source Data Extended Data Fig. 6 Statistical source data.

## Data Availability

The datasets used in this research article can be downloaded from the Gene Expression Omnibus (GEO) accession number GSE231779. Source data are provided with this paper.

## Code availability

Code used to perform the analysis is available at https://github.com/mayer-lab/Dvoretskova-et-al.
