# Supplementary material for: Spatial enhancer activation influences inhibitory neuron identity during mouse embryonic development
Source: Nat Neurosci. 2024 Mar 25;27(5):862–72. doi: 10.1038/s41593-024-01611-9 (PMC11088997; doi:10.1038/s41593-024-01611-9)
Supplement: Supplementary file 1 — Supplementary Tables 1–5. [file 41593_2024_1611_MOESM1_ESM.pdf]

nature neuroscience

Article

https://doi.org/10.1038/s41593-024-01611-9

Spatial enhancer activation influences inhibitory neuron identity during mouse embryonic development

In the format provided by the authors and unedited

Spatial enhancer activation influences inhibitory neuron identity during mouse embryonic development

Supplementary Tables

**sgRNA name**	**Sequence**	**FW**	**RV**	**In vitro ICE KO score (%)**	**In vivo ICE KO score (%)**
gLacZ	GTGCGAATACGCCCACGCGAT	NA	NA	NA	NA
gMeis2	GACGGAGACCCTCACGCGCCG	ATTCGTGTCCTTGGGGAGAAG	ACGACATCCTCTCCCAACTTTC	67	31
gTCF4	GATTCAAGCAATAATGCCCGC	AATAGTTCCCAGACCGGAGC	GGGCCAAAGTAGGGGTAGTG	84	NA
gLhx6	GAGCGGTCCCTTCGAGACAG	CTATCGGCAGTTTTCCAGCG	CATTGTTCAGCGCCGGTTT	71	NA

Supplementary Table 1: **Selected sgRNAs list with primers used to analyze CRISPR interference efficiency.**

**Dataset**	**stage_inj**	**stage_collect**	**guide**	**cell number**	**lineage**	**females**	**embryo**
ED190203	E12.5	P7.5	glacZ	477	no	7	7
			gMeis2	189	no		
			gTcf4	348	no		
			gMkx	347	no		
			gCux2	340	no		
ED191217	E12.5	P7.5	glacZ	7	no	6	6
			gMeis2	59	no		
			gCux2	168	no		
ED200211	E12.5	P7.5	glacZ	836	no	4	4
			gLhx6	836	no		
			gCux2	218	no		
			gMkx	769	no		
ED200218	E12.5	P7.5	glacZ	60	no	4	4
			gLhx6	34	no		
			gMeis2	37	no		
			gTcf4	45	no		
ED200526	E12.5	P7.5	glacZ	3	no	3	3
			gLhx6	834	no		
			gMeis2	289	no		
ED200630	E12.5	P7.5	gCux2	639	no	2	3
			gMeis2	698	no		
ED200804	E12.5	P7.5	glacZ	1246	no	1	2
			gTCf4	6	no		
ED201013	E12.5	P7.5	glacZ	686	no	3	5
			gMkx	4136	no		
			gTcf4	148	no		
ED201006	E12.5	P7.5	glacZ	386	no	3	5
			gMkx	758	no		
ED201020	E12.5	P7.5	gMeis2	254	no	3	5
			gTcf4	1073	no		
ED200806	E12.5	E16.5	glacZ	632	no	2	3
			gMeis2	2598	no		
ED210204	E12.5	E16.5	glacZ	205	yes	2	3
			gMeis2	4161	no		
ED210215	E12.6	E16.6	glacZ	1232	yes	1	1
			gMeis2	53	no	1	1
ED210415	E12.5	E16.5	glacZ	1567	no	2	4
			gMeis2	2175	no		
ED211111	E12.5	E16.5	gMeis2	14485	yes	1	1
ED211124	E12.5	E16.5	glacZ	7373	yes	1	1

Supplementary Table 2: **Datasets information.**

Cluster	Description	Region
Mitotic	Mitotic cells based on high expression of cell-cylce related genes	VZ
IN:Calb2/Nxph1	Precursor of Sst, Pvalb and Th INs	STR, CTX, OB
PN:Tshz1/Pbx3	Precursor of MSNs, ITCs	BNST, AMY
IN:Nr2f2/Nnat	Unable to assign this cluster	N.A.
PN:Foxp1/Six3	Precursor of D2-MSN, Ppp1r1b-type PN	STR, GP
IN:Tiam2/Zfp704	Unable to assign this cluster	N.A.
PN:Foxp1/Isl1	Precursor of MSN	STR
IN:Nfib/Tcf4	Precursor of CGE derived INs	CTX, HC, OB
PN:Isl1/Bcl11b	Precursor of D1-MSN and Pp1r1b-type PN	STR, GP
PN:Ebf1/Zfp503	Precursor of D1-MSN	STR
PN:Meis2/Bcl11b	Precursor of MSN	STR, AMY
IN:Lhx6/Npy	Precursor of MGE derived INs: Pvalb, Sst	CTX, HC
IN:Cck/Reln	Precursor of CGE derived INs: Vip, Reelin	CTX, HC
IN:PN:Isl1/Meis2	Precursor of MSN	AMY, BNST

Supplementary Table 3: **GABAergic precursor clusters and associated brain regions.** The table presents E16 clusters of GABAergic neuronal precursors along with their corresponding descriptions and associated brain regions. At E16, these scRNA-seq clusters represent precursors of adult neuronal types, many of which are in the process of migration to their final settling positions. Due to the ongoing migration and developmental processes, the specific type they will differentiate into and the structure they will migrate to can only be inferred or hypothesized [Mayer et al., 2018, Lee et al., 2022, Lim et al., 2018, Bandler et al., 2022]. We have inferred these potential future fates based on Mousebrain.org [La Manno et al., 2021] and DropViz.org [Saunders et al., 2018]. AMY, Amygdala; BNST, Bed nucleus of the stria terminalis; CGE, caudal ganglionic eminence; MGE, medial ganglionic eminence CTX, Cortex; GP, Globus pallidus; HC, Hippocampus; ITC, intercalated cells; MSN, Medium spiny neuron; D1-MSN, DRD1-expressing MSN (direct pathway striatal projecting neuron); D2-MSN, DRD2-expressing MSN (indirect pathway striatal projecting neuron); Pvalb, Parvalbumin expressing interneuron; OB, Olfactory bulb; Sst, Somatostatin expressing interneuron; Th INs, TH expressing interneuron; Reelin, Reelin expressing interneurons; VIP, VIP expressing interneuron;

sgRNA name	Sequence
P5-R1	5'- AATGATACGGCGACCACCGAGATCTACACTCGTCGGCAGCGTCAGATGTGTATAAGAGACAGTGGTATTTGTGAGCCAGAGTC-3'
P7-N701-Read2	5'- CAAGCAGAAGACGGCATACGAGATGTCAGAAGGTGACTGGAGTTCAGACGTGT-3'
P7-N701-Read4	5'- CAAGCAGAAGACGGCATACGAGATTGGCAAGTGTGACTGGAGTTCAGACGTGT-3'
P7-N701-Read3	5'- CAAGCAGAAGACGGCATACGAGATGGTTCCTTGTGACTGGAGTTCAGACGTGT-3'
P7-N701-Read5	5'- CAAGCAGAAGACGGCATACGAGATCACTGGTTGTGACTGGAGTTCAGACGTGT-3'
P7-N701-Read6	5'- CAAGCAGAAGACGGCATACGAGATCACCTGTAGTGACTGGAGTTCAGACGTGT-3'
P7-N701-Read7	5'- CAAGCAGAAGACGGCATACGAGATGACCACTAGTGACTGGAGTTCAGACGTGT-3'
i501	5'- AATGATACGGCGACCACCGAGATCTACACTAGATCGCACACTCTTTCCCTACACGACGCTCTTCCGATCT-3'
i502	5'- AATGATACGGCGACCACCGAGATCTACACCTCTCTATACACTCTTTCCCTACACGACGCTCTTCCGATCT-3'
i503	5'- AATGATACGGCGACCACCGAGATCTACACTATCCTCTACACTCTTTCCCTACACGACGCTCTTCCGATCT-3'
i504	5'- AATGATACGGCGACCACCGAGATCTACACAGAGTAGAACACTCTTTCCCTACACGACGCTCTTCCGATCT-3'
i505	5'- AATGATACGGCGACCACCGAGATCTACACCAGGACTAACACTCTTTCCCTACACGACGCTCTTCCGATCT-3'
i506	5'- AATGATACGGCGACCACCGAGATCTACACCGCTACTAACACTCTTTCCCTACACGACGCTCTTCCGATCT-3'
i508-lib1	5'- AATGATACGGCGACCACCGAGATCTACACCTAAGCCTACACTCTTTCCCTACACGACGCTCTTCCGATCTGTCCTTCCGAGTGAGAGACAC-3'
i508-lib2	5'- AATGATACGGCGACCACCGAGATCTACACCTAAGCCTACACTCTTTCCCTACACGACGCTCTTCCGATCTTTTGCCCTCCCATATGTCCT-3'
i508-lib3	5'- AATGATACGGCGACCACCGAGATCTACACCTAAGCCTACACTCTTTCCCTACACGACGCTCTTCCGATCTGTCAGATGCTCAAGGGGCTT-3'

Supplementary Table 4: **Primers used to amplify the TrackerSeq library.**

Vector Name	location mm10	size, bp	gene	FW	RV
promoter_Six3	chr17:85,620,500-85,620,950	452	Six3	GTTTCGCCCTCTTCTCCCTC	CACCCCACACACATCCACAT
rev_hs1080	chr6:15,369,186-15,370,104	920	Foxp2	TCTGAAGTCATCGTCTTCCTCAGAATTAG	AGAGTGACTGAAAAGACTCACAAGCT
hs956	chr6:15411327-15413759	2433	Foxp2	ATGCCGCCACTTGGAAGGT	TTGTTTAATAAGGCAGCCCAACACA
hs748	chr14:23,094,641-23,095,790	1149	Zfp503	ACATCATCACCTGAGCCAGC	GGCAGAGGAGTTGAGACAGC
hs1041	chr19:14,776,739-14,777,866	1127	Tle4	TGCAATATGTGGGGCCCATT	CTCCCATCAAAACACGCAGC
enhD1	chr13:53,805,342-53,807,191	1851	Drd1	GCCCGAAGTGTATCCACTTGAAGA	AGGAAACTACCTGCCTGGGG
short enhD1	chr13:53,805,752-53,806,089	331	Drd1	TCGCCGAACAAGCTATGCAC	CGTCTTGCCTTGGTGAGCTTG
enhAldh1a3	chr7:66,492,520-66,493,420	902	Aldh1a3	TTGACTGAGCGATCAAGGAGA	GAGAGATTAGATACAAAGGCACAGC
enhMeis2 rev	chr2:115,725,197-115,726,135	940	Meis2	TGATGGCTGAAAAACCCGGCAC	TTTCTTATCACTGAAGTGGGCCTT
promoter_Tshz1	chr18:84,086,389-84,086,799	492	Tshz1	GATTTTTTGGCCGGTCTTGATCTTCC	CAACGTGCAATCTTTTATGTTATGCTGTA
promoter_PBX3	chr2:34,372,452-34,372,945	495	Pbx3	GCCACCTCCGGTGCC	GCTGGACTGACCGACCATCT
promoter Zfp503	chr14:21,988,271-21,988,966	697	Zfp503	ACTAGGAGTCCTGGGTGC	GGATGAGTGGGAGCCTGTA
hs119	chrX:93,408,898-93,410,235	1339	Arx	GTCCTTCAGGGCAGTTTTCCAAG	TCTTTTCCCCCCTTACCGGAC

Supplementary Table 5: **Information on cloned regulatory elements used in luciferase reporter assays.**

References

[Bandler et al., 2022] Bandler, R. C., Vitali, I., Delgado, R. N., Ho, M. C., Dvoretskova, E., Ibarra Molinas, J. S., Frazel, P. W., Mohammadkhani, M., Machold, R., Maedler, S., Liddelow, S. A., Nowakowski, T. J., Fishell, G., and Mayer, C. (2022). Single-cell delineation of lineage and genetic identity in the mouse brain. *Nature*, 601(7893):404–409.

[La Manno et al., 2021] La Manno, G., Siletti, K., Furlan, A., Gyllborg, D., Vinsland, E., Mossi Albiach, A., Mattsson Langseth, C., Khven, I., Lederer, A. R., Dratva, L. M., Johnsson, A., Nilsson, M., Lönnerberg, P., and Linnarsson, S. (2021). Molecular architecture of the developing mouse brain. *Nature*, 596(7870):92–96.

[Lee et al., 2022] Lee, D. R., Rhodes, C., Mitra, A., Zhang, Y., Maric, D., Dale, R. K., and Petros, T. J. (2022). Transcriptional heterogeneity of ventricular zone cells in the ganglionic eminences of the mouse forebrain. *Elife*, 11.

[Lim et al., 2018] Lim, L., Mi, D., Llorca, A., and Marín, O. (2018). Development and functional diversification of cortical interneurons. *Neuron*, 100(2):294–313.

[Mayer et al., 2018] Mayer, C., Hafemeister, C., Bandler, R. C., Machold, R., Batista Brito, R., Jaglin, X., Allaway, K., Butler, A., Fishell, G., and Satija, R. (2018). Developmental diversification of cortical inhibitory interneurons. *Nature*, 555(7697):457–462.

[Saunders et al., 2018] Saunders, A., Macosko, E. Z., Wysoker, A., Goldman, M., Krienen, F. M., de Rivera, H., Bien, E., Baum, M., Bortolin, L., Wang, S., Goeva, A., Nemesh, J., Kamitaki, N., Brumbaugh, S., Kulp, D., and McCarroll, S. A. (2018). Molecular diversity and specializations among the cells of the adult mouse brain. *Cell*, 174(4):1015–1030.e16.
