# Supplementary material for: Spatial enhancer activation influences inhibitory neuron identity during mouse embryonic development
Source: Nat Neurosci. 2024 Mar 25;27(5):862–72. doi: 10.1038/s41593-024-01611-9 (PMC11088997; doi:10.1038/s41593-024-01611-9)
Supplement: Supplementary file 2 — Reporting Summary [file 41593_2024_1611_MOESM2_ESM.pdf]

nature portfolio

Corresponding author(s): Christian Mayer

Last updated by author(s): Feb 5, 2024

Reporting Summary

Nature Portfolio wishes to improve the reproducibility of the work that we publish. This form provides structure for consistency and transparency in reporting. For further information on Nature Portfolio policies, see our Editorial Policies and the Editorial Policy Checklist.

Statistics

For all statistical analyses, confirm that the following items are present in the figure legend, table legend, main text, or Methods section.

n/a	Confirmed	
☐	☒	The exact sample size (*n*) for each experimental group/condition, given as a discrete number and unit of measurement
☐	☒	A statement on whether measurements were taken from distinct samples or whether the same sample was measured repeatedly
☐	☒	The statistical test(s) used AND whether they are one- or two-sided<br>*Only common tests should be described solely by name; describe more complex techniques in the Methods section.*
☒	☐	A description of all covariates tested
☒	☐	A description of any assumptions or corrections, such as tests of normality and adjustment for multiple comparisons
☐	☒	A full description of the statistical parameters including central tendency (e.g. means) or other basic estimates (e.g. regression coefficient) AND variation (e.g. standard deviation) or associated estimates of uncertainty (e.g. confidence intervals)
☐	☒	For null hypothesis testing, the test statistic (e.g. *F*, *t*, *r*) with confidence intervals, effect sizes, degrees of freedom and *P* value noted<br>*Give P values as exact values whenever suitable.*
☒	☐	For Bayesian analysis, information on the choice of priors and Markov chain Monte Carlo settings
☒	☐	For hierarchical and complex designs, identification of the appropriate level for tests and full reporting of outcomes
☒	☐	Estimates of effect sizes (e.g. Cohen's *d*, Pearson's *r*), indicating how they were calculated

Our web collection on statistics for biologists contains articles on many of the points above.

Software and code

Policy information about availability of computer code

Data collection	Reporter assay: BertholdTech TriStar2S, Driver Version: 1.00 (1.0.0.5), ICE, Version 1.0.9.0. Images were acquired using STELLARIS 5 confocal microscope system (Leica) (Ext. Data Fig. 1c) or LSM 880 laser scanning confocal microscope (Zeiss) (Fig. 3a, Ext. Data Fig. 1b)
Data analysis	The code to reproduce the data analysis is available at https://github.com/mayer-lab/Dvoretskova-et-al<br>Reporter assay: Prism v.10.0.2 .<br>ChIP-seq: Cutadapt (v1.16), Bowtie2 (v2.3.0), Picard (v2.15), MACS2 (v2.1.2), IGV (v2.12.3), HOMER (v4.10.4), SpaMo (v5.4.1)<br>Single-cell transcriptome analyses: Cell Ranger (v3.0.2 or v5.0.1), R (v4.1), R (v3.6), Seurat (4.1.0), Harmony (v1.0), Hotspot (v0.91), enrichR (v3.0), Libra (1.0),<br>For more information see Methods.

For manuscripts utilizing custom algorithms or software that are central to the research but not yet described in published literature, software must be made available to editors and reviewers. We strongly encourage code deposition in a community repository (e.g. GitHub). See the Nature Portfolio guidelines for submitting code & software for further information.

Data

Policy information about availability of data

All manuscripts must include a data availability statement. This statement should provide the following information, where applicable:

- Accession codes, unique identifiers, or web links for publicly available datasets
- A description of any restrictions on data availability
- For clinical datasets or third party data, please ensure that the statement adheres to our policy

The datasets used in this research article can be downloaded from the Gene Expression Omnibus (GEO) accession number GSE231779.

Publically available data used in this study:
from NCBI Gene Expression Omnibus https://www.ncbi.nlm.nih.gov/geo/
GSE167047 (snATAC-seq of E12.5 MGE and LGE)
GSE85705 (LHX6-ChIP-seq GE E13.5)
GSE124936 (DLX1, DLX2 \& DLX5-ChIP-seq GE E13.5)
GSE188528 (scRNA-seq of LGE, MGE, CGE E13.5)
Source data are provided with this paper

Developmental enhancers and interacting genes: Gorkin et. al. (2020), DOI: 10.1038/s41586-020-2093-3
Vista enhancer images were downloaded from the Vista Enhancer browser https://enhancer.lbl.gov
TSS definitions from Eukaryotic Promoter Database (mmEPDnew version 003, https://epd.expasy.org/epd/)
reference genome GRCm38/mm10 was accessed by software as outlined in the Methods

Human research participants

Policy information about studies involving human research participants and Sex and Gender in Research.

Reporting on sex and gender	No human research participants in the study.
Population characteristics	No human research participants in the study.
Recruitment	No human research participants in the study.
Ethics oversight	No human research participants in the study.

Note that full information on the approval of the study protocol must also be provided in the manuscript.

Field-specific reporting

Please select the one below that is the best fit for your research. If you are not sure, read the appropriate sections before making your selection.

☒ Life sciences ☐ Behavioural & social sciences ☐ Ecological, evolutionary & environmental sciences

For a reference copy of the document with all sections, see nature.com/documents/nr-reporting-summary-flat.pdf

Life sciences study design

All studies must disclose on these points even when the disclosure is negative.

Sample size	The sample size was chosen empirically or based on preliminary data to provide a sufficient level of statistical power for detecting indicated biological effects. No statistical methods were used to pre-determine sample sizes, but our sample sizes are similar to those reported in previous publications (Bandler et al. (2022), Nature)
Data exclusions	All data was used for analysis unless apparent failures of experiment.
Replication	Data for in vitro experiments are from three independent experiments, each performed in triplicate. TrackerSeq has n=2 and n=3 replicates per condition. Animals were examined for accurate IUE targeting using a stereoscope. Only the animals with correct targeting were selected for scRNA-Seq (refer to the Methods section for more details).<br>ChIP-seq: GE tissue from 70 embryos was pooled, therefore the data represents an average of these biological replicates. Material was only sufficient for one replicate and input control.
Randomization	We used the same protocol for all samples to perform scRNA-seq. Cell suspensions from multiple brains, including those with different sgRNAs, were pooled to perform multiplexed scRNA-seq. Data analysis followed the same procedure for all groups of different sgRNAs used in this study. Data from different gRNAs were integrated using unsupervised analysis tools. Therefore, randomisation was not used in the tCROPseq experiments. In the analysis part of TrackerSeq, we used a normalisation method to account for clonal patterns using random

shuffling.

Blinding	Human judgement was used only to assess the correct targeting of the IUE. We used clear and simple definitions to determine targeting (see Methods). Blinding was not possible at this stage. Cell suspensions from several brains, including those with different sgRNAs, were pooled to perform multiplexed scRNA-seq, so that different groups were processed in parallel and no human judgement was involved. Data analysis was performed using the same parameters across groups. Treatment conditions were not compared in the immunohistological experiments.

Reporting for specific materials, systems and methods

We require information from authors about some types of materials, experimental systems and methods used in many studies. Here, indicate whether each material, system or method listed is relevant to your study. If you are not sure if a list item applies to your research, read the appropriate section before selecting a response.

Materials & experimental systems

n/a	Involved in the study
☐	☒ Antibodies
☐	☒ Eukaryotic cell lines
☒	☐ Palaeontology and archaeology
☐	☒ Animals and other organisms
☒	☐ Clinical data
☒	☐ Dual use research of concern

Methods

n/a	Involved in the study
☐	☒ ChIP-seq
☒	☐ Flow cytometry
☒	☐ MRI-based neuroimaging

Antibodies

Antibodies used	anti-MEIS1a/MEIS2a, rabbit polyclonal; anti-MEIS2, rabbit polyclonal; both from Mercader et al. (2005), Development; anti-MEIS2 (SCBT, sc-515470-AF594, H-10), anti-LHX6 (SCBT, sc-271433-AF488, A-9), anti-PROX1 (R&D Systems, AF2727), anti-CTIP2 (Abcam, ab18465, 25B6), anti-rabbit AF594 (Invitrogen, A21207); anti-rat AF488 (Invitrogen, A21208); anti-goat AF488 (Invitrogen, A11055)
Validation	anti-MEIS1a/MEIS2a, rabbit polyclonal; anti-MEIS2, rabbit polyclonal (Mercader et al. (2005), Development) were previously used in ChIP-seq studies in the following publications: Penkov et al. (2013), Cell Rep.; Marcos et al. (2015), Development; Delgado et al. (2021), Nat Commun.<br>All other antibodies used in this study were obtained from commercial suppliers and were validated by the manufacturers for their application in immunohistochemistry. The validation is reported on their websites. In addition, the antibodies have been used validated in the literature:<br>anti-MEIS2 (SCBT, sc-515470-AF594): e.g. PMIDs: 35781337, 29928868<br>anti-LHX6 (SCBT, sc-271433-AF488) e.g. PMIDs: 37254876, 36583474<br>anti-PROX1 (R&D Systems, AF2727) e.g. PMIDs: 36033614, 37224811<br>anti-CTIP2 (Abcam, ab18465) e.g. PMID: 38025769

Eukaryotic cell lines

Policy information about cell lines and Sex and Gender in Research

Cell line source(s)	Mouse Neuro2a neuroblastoma cells (ECACC, 89121404).
Authentication	The cell line was not authenticated.
Mycoplasma contamination	The cell line was not tested for mycoplasma.
Commonly misidentified lines (See ICLAC register)	We did not use misidentified cell lines.

Animals and other research organisms

Policy information about studies involving animals; ARRIVE guidelines recommended for reporting animal research, and Sex and Gender in Research

Laboratory animals	Adult mice were used for breeding, and their embryos at e14.5, e16.5 and pups at P7 for brain tissue collection. Wild type C57BL/6 and CAS9-EGFP (B6.Gt(ROSA)26Sortm1.1(CAG-cas9*,-EGFP)Fezh/J, Jax 026179) mouse lines were used. Mice were group housed in isolated ventilated cages (room temperature 22±1°C, relative humidity 55±5%) under a 12h dark/light cycle with ad libitum access to food and water.
Wild animals	No wild animals were used in the study.

Reporting on sex	The sex was not considered in the study.
Field-collected samples	No field-collected samples were used in the study.
Ethics oversight	CNIC Ethics Committee, Spanish laws, and the EU Directive 2010/63/EU. Animal Protocol: ROB-55.2-2532.Vet_02-18-81 from the government of Upper Bavaria for the Max Planck Institute for Biological Intelligence. ROB-55.2-2532.Vet_02-20-199 from the government of Upper Bavaria for the Helmholtz Zentrum München.

Note that full information on the approval of the study protocol must also be provided in the manuscript.

ChIP-seq

Data deposition

☒ Confirm that both raw and final processed data have been deposited in a public database such as GEO.

☒ Confirm that you have deposited or provided access to graph files (e.g. BED files) for the called peaks.

Data access links<br>*May remain private before publication.*	GEO accession number GSE231779 (secure reviewer access token: cpclgssspnqpvmv)<br>https://github.com/mayer-lab/Dvoretskova-et-al
Files in database submission	IP_GE2_ChIPSeq_S74_L007_R1_001.fastq.gz ; Input_GE2_ChIPSeq_S73_L007_R1_001.fastq.gz;<br>GE_meis2_IP_q0.01_peaks.narrowPeak ; GE_meis_IP_q0.01_treatment_pileup.bw ; GE_meis_IP_q0.01_control_lambda.bw
Genome browser session<br>(e.g. UCSC)	https://genome.ucsc.edu/s/anon_user_17/MEIS_GE_ChIP%2Dseq_mm10

Methodology

Replicates	No ChIP-seq replicates were performed; Chromatin was isolated from 70 wt embryos, PFA-fixed and subsequently pooled before further processing.
Sequencing depth	Total reads IP: 145458809; uniquely mapped reads IP: 98343346 ; Total reads input: 128801698; uniquely mapped reads input: 88651076; 61bp single-end reads were sequenced.
Antibodies	anti-MEIS1a/MEIS2a, rabbit polyclonal; anti-MEIS2, rabbit polyclonal; both from Mercader et al. (2005), Development
Peak calling parameters	macs2 callpeak -t /GE_meis_IP_mm10_sorted_rmdup.bam -c /GE_meis_input_mm10_sorted_rmdup.bam -n GE_meis_IP_q0.01 -B -f BAM -g mm -q 0.01 --/called_peaks/
Data quality	We used 1% FDR as cutoff, and detected 3807 peaks with an FDR<1%, of which 2514 had above 5-fold enrichment. Strand cross-correlation analysis by Phantompeakqualtools yielded NSC=1.05 and RSC=3.1.
Software	reads were trimmed using Cutadapt (v1.16) and mapped to mm10 using Bowtie2 (v2.3.0). Duplicates were removed using Picard (v2.15.0), followed by peak calling with MACS2 (v2.1.2) using a cutoff of q=0.01.
